# Preparation of Novel Nitrogen-Rich Fluorinated Hyperbranched Poly(amide-imide) and Evaluation of Its Electrochromic Properties and Iodine Adsorption Behavior

**DOI:** 10.3390/polym15234537

**Published:** 2023-11-25

**Authors:** Zebang Sun, Wen Yang, Xiaosa Zhang, Xiaoyu Zhu, Jian Luan, Wenze Li, Yu Liu

**Affiliations:** 1 College of Science, Shenyang University of Chemical Technology, Shenyang 110142, China; sunzebang0826@163.com (Z.S.); wenyang120105@163.com (W.Y.); xszhang@syuct.edu.cn (X.Z.); zhuxiaoyu@syuct.edu.cn (X.Z.); 2 College of Sciences, Northeastern University, Shenyang 110819, China; 2010044@stu.neu.edu.cn

**Keywords:** hyperbranched, nitrogen-rich, electrochromic, poly(amide-imide)

## Abstract

In this study, we successfully synthesized a novel triacid monomer by means of the thermal cyclization reaction. Subsequently, a series of nitrogen-rich (A_3_+B_2_)-type fluorinated hyperbranched poly(amide-imide)s (denoted as PAI-1 and -2, respectively) were prepared by means of a one-pot method using this triacid monomer and a diamine monomer with a triphenylamine-carbazole unit as precursors. The degree of support of the prepared hyperbranched PAIs was found to be about 60% via ^1^H NMR calculations. Through X-ray photoelectron spectroscopy (XPS), it was found that the binding energies of C-N (398.4 eV) and -NH (399.7 eV) became lower under a current, while the binding energy peak of N^+^ appeared at 402.9 eV. In addition, the PAIs have good solubility and thermal stability (*T_g_*s: 256–261 °C, T_10%_: 564–608 °C). Cyclic voltammetry (CV) analysis shows that the hyperbranched PAI films have good redox properties, and a range of values for the HOMO (4.83 to 4.85 eV) versus LUMO (1.85 to 1.97 eV) energy levels are calculated. The PAI films have excellent electrochromic properties: PAI-1 on coloration efficiency (CE) and transmittance change (ΔT, 852 nm) are 257 cm^2^/C and 62%, respectively, and have long-lasting redox properties (100 cycles). In addition, we conduct iodine adsorption tests using the structural features of PAIs with electron-drawing units, and the results show that PAI-1 had a high adsorption capacity for iodine (633 mg/g).

## 1. Introduction

Fluorinated hyperbranched polymers (HBPs) are a class of polymeric materials with highly branched structures which distinguish them from traditional linear polymers [1,2,3,4,5]. HBPs have several significant advantages, including (1) good solubility, which facilitates the preparation of polymer films [6,7,8,9]; (2) high intrinsic thermal stability [10,11,12,13]; and (3) molecular structural diversity, which are molecularly designed to make the polymers multifunctional [14,15], with capabilities such as electrochromic behavior [16,17,18,19], iodine adsorption capabilities [20,21], or other desirable properties. These unique properties provide HBPs with a broad application prospects.

HBPs with diphenylamine/triphenylamine-based structures have two key advantages. Firstly, N atoms migrate from the outermost orbital electrons under the excitation of an electric current to form the radical cation N^+^, which causes these polymers to exhibit obvious color changes [22,23,24,25]. Secondly, N atoms have an electron-absorbing effect due to their outer lone pair of electrons, which enhances the adsorption of iodine [26,27]. For example, Zhang et al. prepared polymer films with excellent electrochromic properties (optical contrast: 72.8%; tinting efficiency: 136.26 cm^2^/C) using arylboronic esters and triphenylamino arylbromides as polymerization monomers [28]. Similarly, Zheng et al. designed and prepared electrochromic polymers with triphenylamine units with high self-healing properties (optical contrast: 42.7%; tinting efficiency: 108.3 cm^2^/C, self-healing rate: 80%), which solved the problems of electrochromic deterioration and rupture susceptibility [29]. In addition, Wang et al. simultaneously introduced triphenylamine units and imine bonds into the polymer to enhance the iodine adsorption capabilities (4010–4580 mg/g) [30].

In our previous research work, a variety of nitrogen-rich (A_3_+B_2_)-type fluorinated hyperbranched polymers were designed and prepared, including poly(ester imides) (PEIs) [31], polyazomethines (PAMs) [32], polyamides (PAs) [33], hyperbranched polyimides (HBPIs) [34,35], and an azo-link polymer (Azo-Car-CF_3_) [36]. The results obtained so far can be summarized as follows: (1) all prepared polymers have good solubility (e.g., *N*-methylpyrrolidone (NMP), *N,N*-dimethylacetamide (DMAc), *N,N*-dimethylformamide (DMF)) and thermal stability (T_10%_ ≈ 600 °C); (2) all prepared polymers have good optical properties and color variability (CE: 191–203 cm^2^/C); and (3) the electron-withdrawal effect of nitrogen atoms and trifluoromethyl groups played an active role in iodine adsorption (1198 mg/g).

Based on the above considerations, our studies were aimed at the color-changing behavior of N atoms that could be oxidized to the radical cation N^+^ under the action of an electric current and the adsorption of iodine via the electron-withdrawal effect of N and F atoms. To this end, we designed and synthesized a novel fluorinated triacid monomer and characterized its structure in detail. Subsequently, a series of nitrogen-rich (A_3_+B_2_)-type fluorinated hyperbranched poly(amide-imide)s (individually denoted as PAI-1 and PAI-2) were prepared by means of a one-pot method using this triacid monomer and a diamine monomer with a triphenylamine-carbazole unit as precursors. Subsequently, we characterized the structures and properties of the prepared polymers. The results show that these polymers have a high degree of branching, solubility, thermal stability and good electrochromic properties. In addition, we also investigated the adsorption of iodine using our polymers which contain N and F, which have electron-absorbing properties.

## 2. Results and Discussion

### 2.1. Structural Characterization of Triacid Monomer (BTTFTDCA) and Pendant Monomers

**Figure 1:**
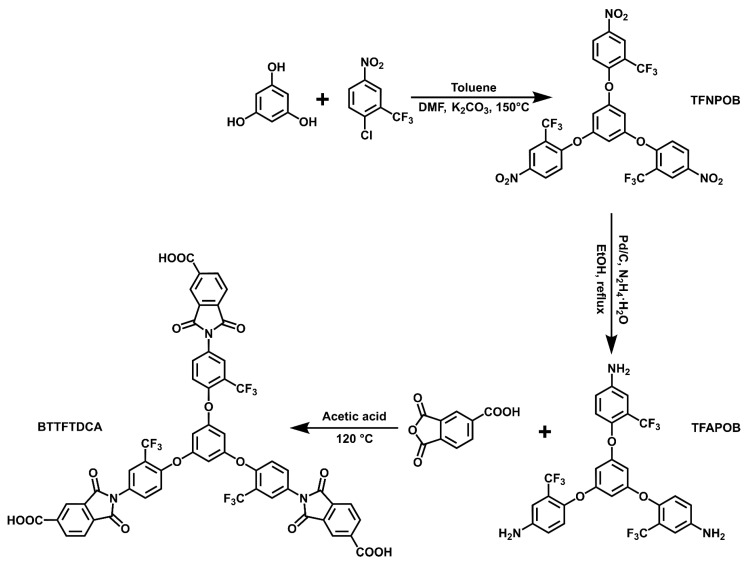
Synthesis route towards the triacid monomer BTTFTDCA.

As described in this section, we conducted structural characterization of the newly prepared triacid monomers. The triacid monomer precursor, 1,3,5-tris(4-amino-2-(trifluoromethyl)phenoxy)-benzene (TFAPOB), and diamine monomers, 3″-trifluoromethyl-4,4′-diamino-4″-N-carbazolyl triphenylamine (TCA-F) and 4,4′-bis[(4-aminophenyl)amino]-4″-carbazolyltriphenylamine (TCIA), were prepared following the previously reported literature [32,33,37]. Detailed information regarding their preparation process (see in Supplementary Material Schemes S1–S3) and structural characterization (see in Supplementary Material Figures S1–S6) is shown in the Supporting Information. Figure 1 illustrates the synthesis of the triacid precursor TFAPOB through a coupling reaction and hydrazine hydrate reduction reaction using *m*-phenyltriol and 2-chloro-5-nitrobenzotrifluoride as reactive monomers. Subsequently, the triacid monomer BTTFTDCA was obtained through a thermal cyclization reaction of the triamine monomer (TFAPOB) and trimellitic anhydride in acetic acid solvent.

The FT-IR, ^1^H NMR, ^13^C NMR, and XRD analyses were performed to confirm the structure of the triacid monomer. In the FT-IR spectrum (see in Supplementary Material Figure S7), the disappearance of the –NH_2_ stretching vibration (3508 cm^−1^, 3411 cm^−1^), originally attributed to the triamine monomer, was observed after the reaction with trimellitic anhydride. Vibrational peaks corresponding to hydroxyl groups on the carboxyl group appeared between 3428 and 2533 cm^−1^. Meanwhile, C=O vibrational peaks were observed at 1702 cm^−1^, and C–N vibrational peaks belonging to the imine ring appeared at 1285 cm^−1^ [38,39].

**Figure 2:**
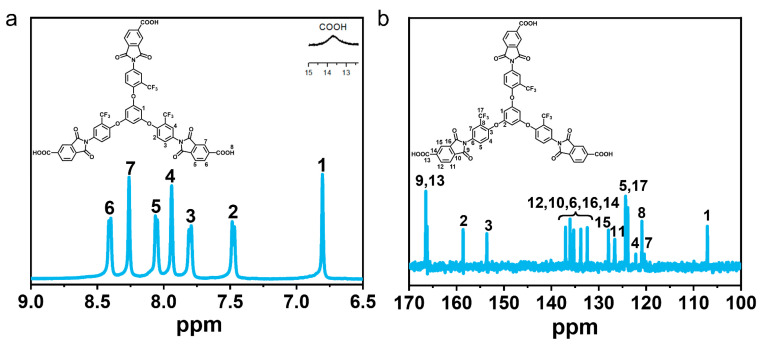
(**a**) ^1^H NMR spectrum of BTTFTDCA recorded in DMSO-*d*_6_; (**b**) ^13^C NMR spectrum of BTTFTDCA recorded in DMSO-*d*_6_.

The ^1^H NMR spectra of the triacid monomers (Figure 2a) showed that the signal for hydrogen atom labeled as No. 1 appeared in the highest field, indicating it was not surrounded by any electron-absorbing groups, while the signal for hydrogen atom No. 8 appeared in the lowest field as the active hydrogen signal. Additionally, the structure of the triacid monomer was confirmed via ^13^C NMR spectroscopy (Figure 2b). The ^13^C NMR signal of the carbon atom labeled as No. 1 in Figure 2b appeared in the highest region (107.2 ppm), whereas the signal of carbon atom No. 9 of the imide ring unit and carbon No. 13 on the carboxylate group appeared in the lowest field region (166.4 ppm) due to the effect of its electron-withdrawing nature.

### 2.2. Structural Characterization of Poly(amide-imide) (PAI-1 and PAI-2)

**Figure 3:**
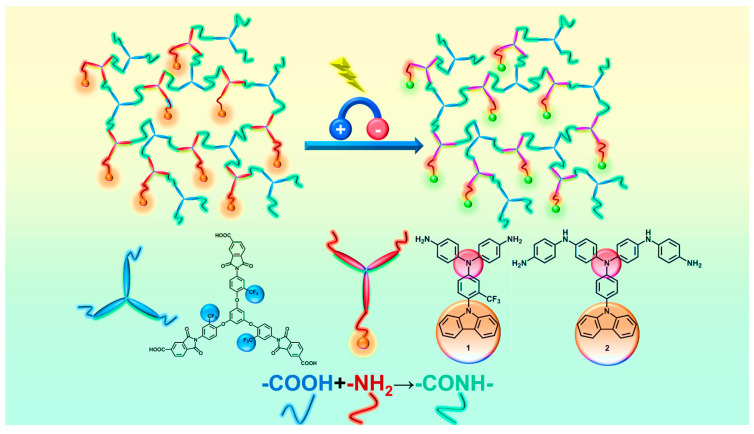
The preparation flowchart for PAI-1 and PAI-2.

In this section, we successfully synthesized and characterized two hyperbranched poly(amide-imide) (PAI) polymers with pendant units and electrochromic properties, with the feedstocks involved in the preparation being BTTFTDCA, TCA-F, and TCIA (Figure 3 and Supplementary Material Scheme S4).

**Figure 4:**
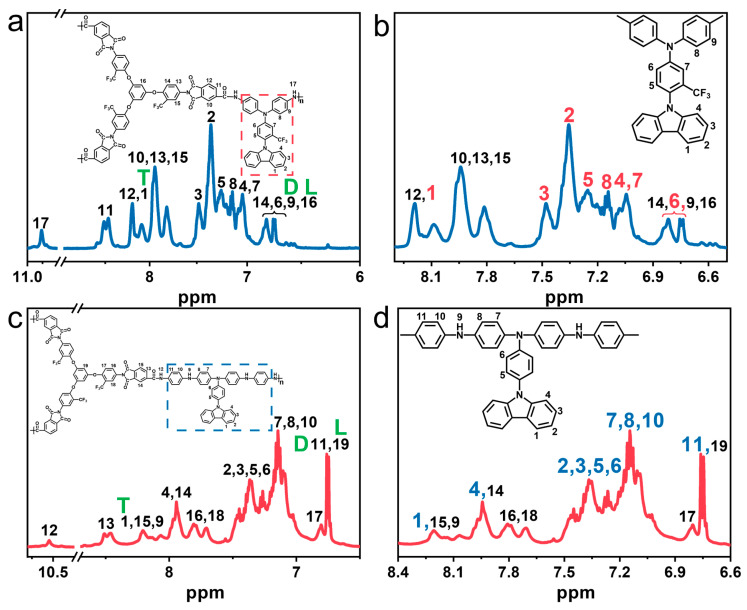
(**a**,**b**) ^1^H NMR spectra of PAI-1 recorded in DMSO-*d*_6_; (**c**,**d**) ^1^H NMR spectra of PAI-2 recorded in DMSO-*d*_6_.

Figure 4 displays the ^1^H NMR spectra of PAI-1 and PAI-2. The signals corresponding to the hydrogen atoms labelled as No. 17 in PAI-1 and labelled as No. 12 PAI-2, which are part of the –CO–NH- group, appeared at the lowest field, with chemical shifts of 10.77 ppm (No. 17) and 10.54 ppm (No. 12), respectively (Figure 4a,c). This can be attributed to the strong electron absorption effect of the carbonyl group. Additionally, the signals for hydrogen atoms No. 6, No. 9, No. 14 and 16 of PAI-1, as well as hydrogen atoms No. 11 and 19 of PAI-2, appeared at the highest field due to the absence of electron-absorbing groups in their vicinity. Meanwhile, the hydrogen atoms labelled as No. 1 to 9 of PAI-1 and hydrogen atoms No. 1 to 11 of PAI-2 were assigned to the diamine units of TCA-F and TCIA, respectively (Figure 4b,d).

(1) $$DB=\frac{2D}{D+L+T}$$

In addition, ^1^H NMR spectroscopy is also an effective method for determining the degree of branching (*DB*) in hyperbranched polymers [40,41]. By using ^1^H NMR spectroscopy, the integral areas of the absorption peaks corresponding to the branched unit, linear unit, and end group unit can be obtained, which represent the content of branched modular (*D*), linear modular (*L*), and end group modular (*T*) in the hyperbranched polymer. The degree of polymer branching can be calculated by combining these values with Equation (1). Taking hyperbranched PAI-1 as an example, the absorption peak positions for *D*, *L*, and *T* were observed at 6.74 ppm, 6.76 ppm, and 8.08 ppm, respectively (Figure 4a). The degree of branching (DB) in PAI-1, calculated according to Equation (1), was found to be 60%. Similarly, PAI-2 exhibited a branching degree of 61% (Figure 4c).

**Figure 5:**
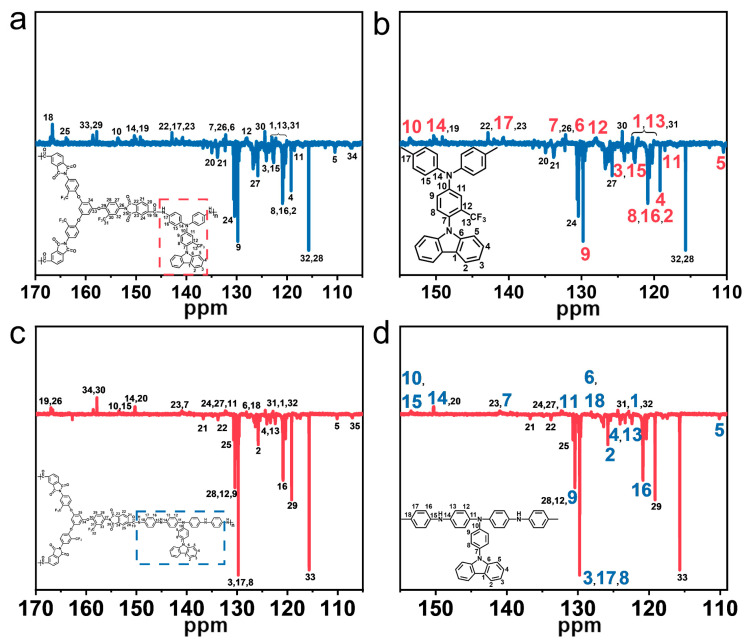
(**a**,**b**) ^13^C-Dept NMR spectra of PAI-1 recorded in DMSO-*d*_6_; (**c**,**d**) ^13^C-Dept NMR spectra of PAI-2 recorded in DMSO-*d*_6_.

Moreover, we employed ^13^C-Dept NMR spectroscopy to further characterize the chemical structures of PAI-1 and PAI-2 (Figure 5). The signals for carbon atom No. 18 of PAI-1 and carbon atoms No. 19 and 26 of PAI-2, corresponding to the imide ring, were observed at the lowest field due to the strong electron absorption effect of the carbonyl and nitrogen atoms. Similarly, carbon atom No. 34 of PAI-1 and carbon atom No. 35 of PAI-2 appeared at the highest field, likely resulting from the absence of surrounding electron-absorbing groups (Figure 5a,c). Additionally, carbon atoms No. 1 to 17 of PAI-1 and carbon atoms No. 1 to 18 of PAI-2 were assigned to the diamine units TCA-F and TCIA, respectively (Figure 5b,d).

The ^1^H NMR and ^13^C-Dept NMR analyses confirmed the successful synthesis of hyperbranched PAI-1 and PAI-2, with the intended incorporation of the pendant units into their structures.

**Figure 6:**
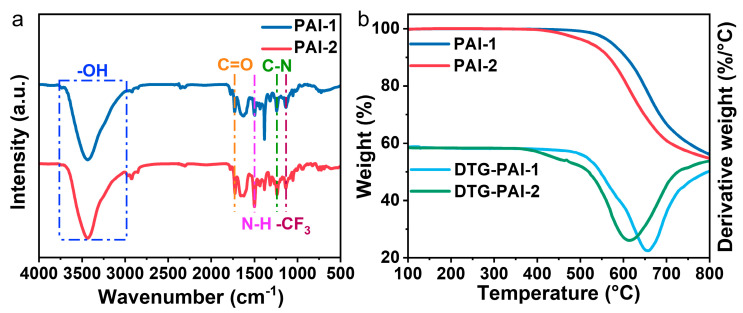
(**a**) FT-IR spectra of PAI-1 and PAI-2; (**b**) TGA and DTG plots of PAI-1 and PAI-2.

After analyzing the NMR spectra of hyperbranched PAIs, we proceeded to analyze them using FT-IR and UV–Vis spectroscopy. Figure 6a shows the FT-IR spectra of these PAIs, with the –OH stretching peak being located at 3759~2978 cm^−1^ and belonging to the terminal carboxyl group, while the C=O stretching peak appeared at 1730 cm^−1^; the N–H stretching peak, which belonged to the –CONH– group, appeared at 1501 cm^−1^; the C–N stretching peak, which belonged to the triphenylamine unit, appeared at 1241 cm^−1^; and lastly, the –CF_3_ stretching appeared at 1131 cm^−1^, as before. The FT-IR spectra show that the preparation of PAI-1 and PAI-2 was successful, and the original structures of the monomers were not destroyed during the polymerization reaction. We then tested the optical properties of PAI via UV-Vis spectroscopy, as shown in Supplementary Material Figure S8, where these polymers were dissolved in NMP and the measured absorption peaks were in the range of about 337 nm. In addition to this, all three polymers showed amorphous structures according to the XRD characterization of these three hyperbranched PAIs (see in Supplementary Material Figure S9). The introduction of pendant units and trifluoromethyl disrupts the regular arrangement between molecules and the process becomes easier due to the presence of flexible ether bonds. Meanwhile, this disordered arrangement increases the solubility of the polymers and provides greater ease in the preparation of thin films.

### 2.3. Thermal Properties of the PAIs

As shown in Figure 6b, the thermal stability of the PAIs was evaluated using thermogravimetric analysis (TGA) in a nitrogen atmosphere with temperatures at 5% and 10% weight loss as reference points. The decomposition temperatures of the PAIs at 5% weight loss were in the range of 514 to 574 °C, and those at 10% weight loss were in the range of 564 to 608 °C. In addition, the charring rate of these polymers at 800 °C measured under a nitrogen atmosphere was more than 55%, which indicates that they have excellent thermal stability. In addition, derivative thermogravimetric (DTG) analysis was used to further analyze the decomposition process of the polymers. All three polymers had only one peak transition process, all after 500 °C, thus further demonstrating the good thermal stability of the two hyperbranched PAIs. As summarized in Table 1, all three polymers show high glass transition temperatures (256–261 °C). The high degree of branching allows the polymers to maintain their inherent thermal stability, and this excellent thermal stability provides the polymers with potential for research in high-temperature applications [42,43].

### 2.4. Inherent Viscosity, GPC Value and Solubility of the PAIs

The molecular weight (*M*_w_) of the obtained products and their polydispersities are presented in Table 2, with the range spanning from 62,000 to 65,000 g/mol and 2.3 to 2.1, respectively. The intrinsic viscosity of the PAIs was determined at 25 °C in DMAc using an Ubbelohde viscometer. The obtained values varied within the range of 0.41 to 0.43 dL g^−1^. The solubility of the prepared hyperbranched PAIs1–2 in a variety of organic solvents is summarized in Table 2 (consistently at 1.0% (*w*/*v*)). The PAIs demonstrated good solubility in solvents such as NMP, DMAc, DMF, and DMSO. This solubility can be attributed to the introduction of capping monomers into the polymers, which prevents the tight stacking between the polymer molecular chains, while the regular intermolecular arrangement is disturbed due to the presence of capping units and trifluoromethyl groups, making the polymers more soluble. In addition to this, the large number of flexible ether bonds in the structure helps to improve the solubility of the polymer. Consequently, these polymers are highly processable and have a promising future for practical applications relying on spin- or dip-coating processes, and this outcome is consistent with the XRD data [44].

### 2.5. Film Forming Properties of the PAIs

A homopolymer solution was prepared by adding 1.0 g of the PAI sample to 10 mL of NMP. A glass dish with a diameter of 4 cm was used as a vessel into which the solution was added, and the solution was then dried overnight in an oven at 90 °C to remove most of the NMP. Subsequently, the film was removed and further dried in a vacuum oven at 180 °C for 5 h. The prepared polymer films had thicknesses in the range of 71–75 microns. The prepared hyperbranched PAI-1 and PAI-2 had a root mean square (RMS) roughness of 0.11 and 0.13 nm and maximum roughness of about 1.9 and 1.7 nm, respectively, which were found to be smooth and continuous according to the atomic force microscopy (AFM) images of the polymers (see in Supplementary Material Figures S10 and S11).

### 2.6. Electrochemical Characteristics

**Figure 7:**
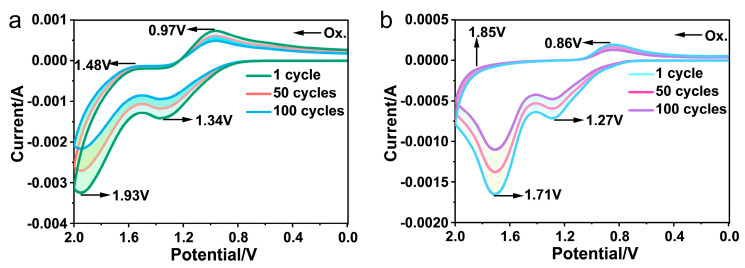
(**a**) Repeated CV scans of PAI−1 films on the ITO−coated glass substrate in 0.1 M Bu_4_NClO_4_ at a scan rate of 100 mV/s; (**b**) Repeated CV scans of PAI−2 films on the ITO−coated glass substrate in 0.1 M Bu_4_NClO_4_ at a scan rate of 100 mV/s.

As described in this section, the electrochemical properties of PAIs were assessed (100 cycles) via cyclic voltammetry (CV). We conducted electrochemical performance testing on the PAI film using cyclic voltammetry (CV) in a three-electrode electrochemical cell. The PAI/ITO glass served as the working electrode (length: 2 cm, width: 0.5 cm), a platinum wire (Pt) served as the counter electrode, and Ag/AgCl served as the reference electrode. The preparation process was as follows: the PAI solutions were drop coated onto conductive ITO glass under nitrogen protection. After drying, the coated ITO glass was placed as a working electrode in a solution containing anhydrous acetonitrile (CH_3_CN) and 0.1 mol of tetrabutylammonium perchlorate (TBAP) electrolyte. The obtained CV curves for the PAIs are depicted in Figure 7, revealing that each of the two PAIs displayed a series of reversible redox peaks during anodic scanning. Specifically, the incorporation of triphenylamine and carbazole units led to the emergence of two pairs of redox peaks in the cyclic voltammetry curves. The half-wave potentials of the redox peaks for PAI-1 were observed at 1.16 (*E*_ox1_ 1/2) and 1.71 V (*E*_ox2_ 1/2), respectively. Similarly, the redox peaks for PAI-2 appeared at 1.07 (*E*_ox1_ 1/2) and 1.78 V (*E*_ox2_ 1/2), respectively. The redox peaks represent the electrochemical activity and stability of the polymers.

The energy levels of the highest occupied molecular orbital (HOMO) and the lowest unoccupied molecular orbital (LUMO) corresponding to PAIs were calculated using the onset of oxidation (*E*_onset_) and half-wave potential (*E*_1/2_) values obtained in cyclic voltammetry (CV) tests. The calculations proceeded as follows: the *E*_onset_ value for polymer PAI-1 was measured to be 0.61 V. Additionally, the external redox standard *E* (Fc/Fc^+^) value for ferrocene/ferrocenium (Fc/Fc^+^) was 0.58 V relative to Ag/AgCl in CH_3_CN. Assuming a HOMO energy of 4.80 eV for the Fc/Fc^+^ standard (with reference to the zero-vacuum level), the HOMO and LUMO energy level of PAI-1 could be determined. Consequently, assuming a HOMO energy of 4.80 eV for the Fc/Fc^+^ standard (referenced at the zero-vacuum level), the corresponding HOMO and LUMO energy levels for PAI-2 could be established. The HOMO (4.83 eV) and LUMO energies (1.78 eV) of PAI-1 were calculated, and the properties of PIA-2 are also listed in Table 3 ((1) *HOMO* = *E_onset_* + 4.80 − *E_(Fe/Fe+)_* (*E_onset_*: starting potential; 4.80: value of the HOMO energy level relative to the zero-degree-vacuum energy level; *E_(Fe/Fe+)_*: the standard potential for ferrocene redox); (2) *LUMO* = *HOMO* − *E_g_* (*E_g_* = 1240/*λ_onset_*)). Due to the high HOMO energy level and reversible electrochemical oxidation of these PAIs, they are promising candidates for application in organic light emitting devices (OLEDs).

### 2.7. Electrochromic Characteristics

**Figure 8:**
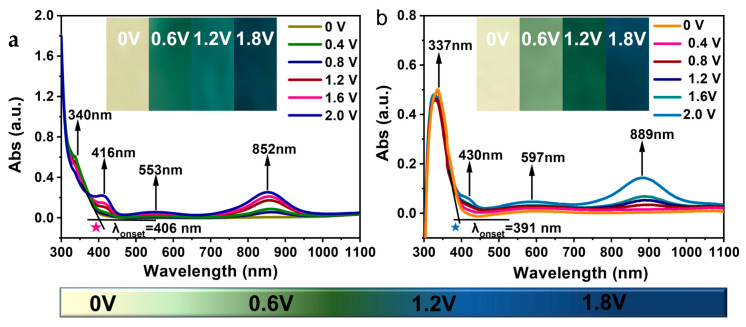
(**a**) Spectroelectrochemical responses of PAI-1 cast films on ITO-coated glass substrates at different applied potentials and electrochromic phenomena of PAIs films in 0.1 M Bu_4_NClO_4_ solution; (**b**) Spectroelectrochemical responses of PAI-1 cast films on ITO-coated glass substrates at different applied potentials and electrochromic phenomena of PAIs films in 0.1 M Bu_4_NClO_4_ solution.

**Figure 9:**
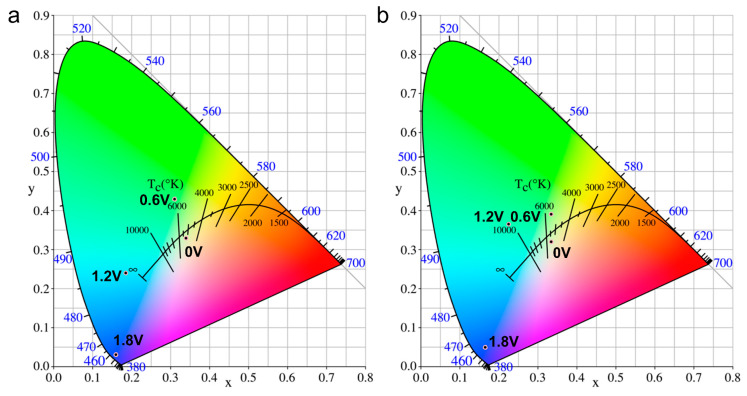
CIE chromaticity diagrams corresponding to the color changes exhibited by (**a**) PAI-1 and (**b**) PAI-2 films at different voltages.

The optically transparent thin layer electrodes (OTTLE) and UV-visible spectrophotometry were used to evaluate the electrochromic properties of our PAI films. Refer to the cyclic voltammetry (CV) test for the relevant test methods. The specific test process was as follows: at 0 V voltage, using the ITO conductive glass sample of the load film as the initial sample, taking the electrochromic behavior generated by applying 0.2 V voltage as an example, setting the operating voltage to 0.2 V, and applying continuous 4 s voltage to the polymer film, the color of the polymer film changes. At this time, the UV-visible spectrum test is performed on the discolored film. In addition, the current change within these 4 s is shown in Supplementary Material Figure S12. Electrochromic testing at other operating voltages and so on. Figure 8 shows the electrochromic absorption spectra of the PAI-1 and PAI-2 films. Three new bands emerge at 416, 553, and 852 nm for PAI-1 and 430, 597, and 889 nm for PAI-2. In particular, the absorption peaks at 852/899 nm are more pronounced, attributed to the presence of triphenylamine and carbazole units in the polymers. Additionally, the PAI-1 and PAI-2 films exhibited color changes from their initial yellowish neutral state (PAI-1: x = 0.345, y = 0.325; PAI-2: x = 0.346, y = 0.319) to yellowish-green (PAI-1: x = 0.324, y= 0.431; PAI-2: x = 0.347, y = 0.389) to blue-green (PAI-1: x = 0.181, y = 0.242; PAI-2: x = 0.234, y = 0.371), and finally to a dark blue (PAI-1: x = 0.161, y = 0.031; PAI-2: x = 0.172, y = 0.053). The chromaticity changes in the fully oxidized state of PAIs films were recorded using CIE-1931 as a standard (Figure 9), and the optical image of the reversible electrochromism of PAIs is recorded in Supplementary Material Figure S13.

**Figure 10:**
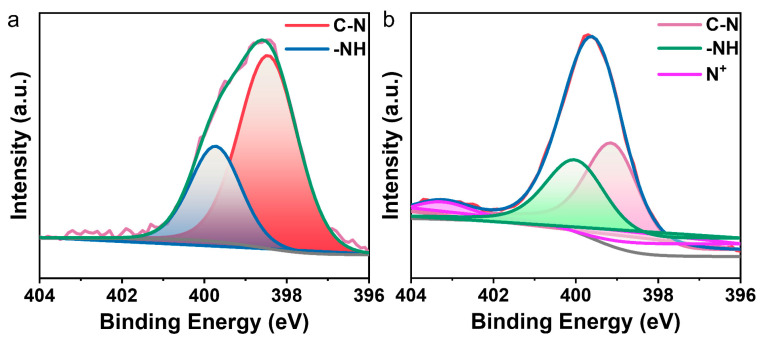
(**a**) XPS spectrum of elemental N prior to the electrochromic transition of PAI-2; (**b**) XPS spectrum of elemental N after the electrochromic transition of PAI-2.

The energy variation of elemental N during the electrochromic transitions of polymers was further investigated via XPS. Taking PAI-2 as an example, Figure 10a shows the binding energies of elemental N when the polymer did not undergo an electrochromic transition, with the C-N binding energy appearing at 398.4 eV and the -NH binding energy appearing at 399.7 eV. After the electrochromic test, the binding energies of C-N and -NH decrease, and the binding energy peak of the radical cation N^+^ appears at 402.9 eV (Figure 10b). Through XPS, the generation of N^+^ was verified, further validating the mechanism of the polymer’s electrochromic behavior [45].

**Figure 11:**
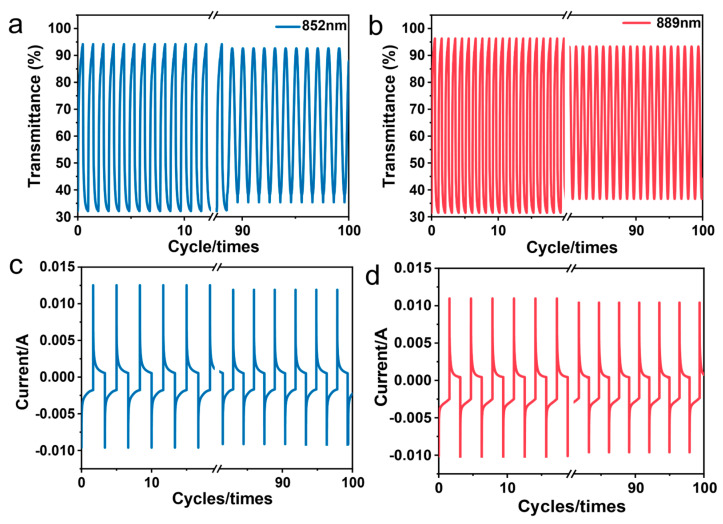
(**a**,**b**) Variation of transmittance of PAI-1 and PAI-2 films on ITO-coated glass substrates; (**c**,**d**) variation of current density of PAI-1 and PAI-2 films on ITO-coated glass substrates in 0.1 M Bu_4_NClO_4_/CH_3_CN solution for switching studies.

We analyzed the electrochromic properties of PAI-1 and PAI-2 films by measuring the change in absorption intensity at wavelengths of 852 nm and 889 nm, respectively. The voltage was continuously switched between 0 V in the neutral state and 2.0 V in the fully oxidized state. In addition, in order to obtain a faster response, we reduced the thickness of the film. The transmittance at specific wavelengths was measured via the UV-Vis-NIR method during the tests. Figure 11a,b depict the transmittance changes of the PAI films at 852 and 889 nm during the first 200 s, as well as the percentage difference between 0.0 and 2.0 V. The transmittance of the PAIs was monitored throughout the first 100 cycles at these wavelengths. In these measurements, the electrochromic contrast (Δ*T*) values for PAI-1 and PAI-2 films at 852 nm and 889 nm were 62% and 66%, respectively, and we can see that the polymer films have only about 6% and 8% degradation after many cycles, which proves that the polymerization has good cycling stability. Furthermore, both the PAI-1 and PAI-2 films demonstrated excellent electrochemical and electrochromic reversibility, even after cycling between 0.0 and 2.0 V for 100 cycles, as shown in Figure 11c,d.

**Figure 12:**
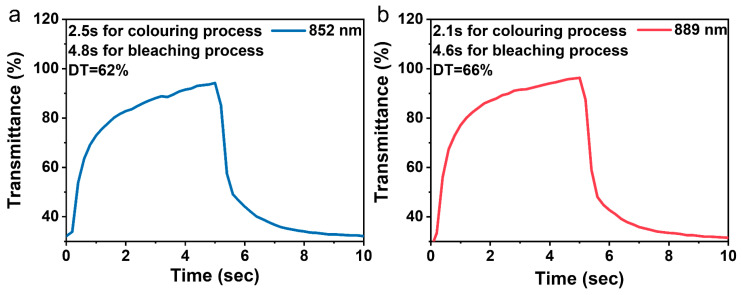
Transmittance profiles of (**a**) PAI-1 and (**b**) PAI-2 films cycled at the indicated wavelengths in 0.1 M Bu_4_NClO_4_/CH_3_CN solution.

The transmittance variation of thin films of PAI-1 and PAI-2 in one cycle is as follows: as shown in Figure 12, the transmittance of thin films of PAI-1 and PAI-2 changes at wavelengths of 852 and 889 nm, respectively. The duration of this transmittance change for thin films of PAI-1 and PAI-2 at these specific wavelengths is 10 s, respectively. The coloring times of thin films of PAI-1 and PAI-2 at 852 nm and 889 nm are 2.5 s and 2.1 s (during oxidation), while the fading times during reduction at a reduction voltage of 0 V are 4.8 s and 4.6 s, respectively. The electrochromic efficiency (CE) can be calculated using Equation (2):

(2) $$CE=\frac{∆OD}{Q}$$

where Δ*OD* represents the change in optical absorbance and *Q* (mC/cm^2^) signifies the charge injected/released during the redox process. Applying this equation, the *CE* values of the PAI-1 and PAI-2 films were determined as 257 and 242 cm^2^/C at 852 nm and 889 nm, respectively.

### 2.8. Adsorption of Iodide Ion Solutions

**Figure 13:**
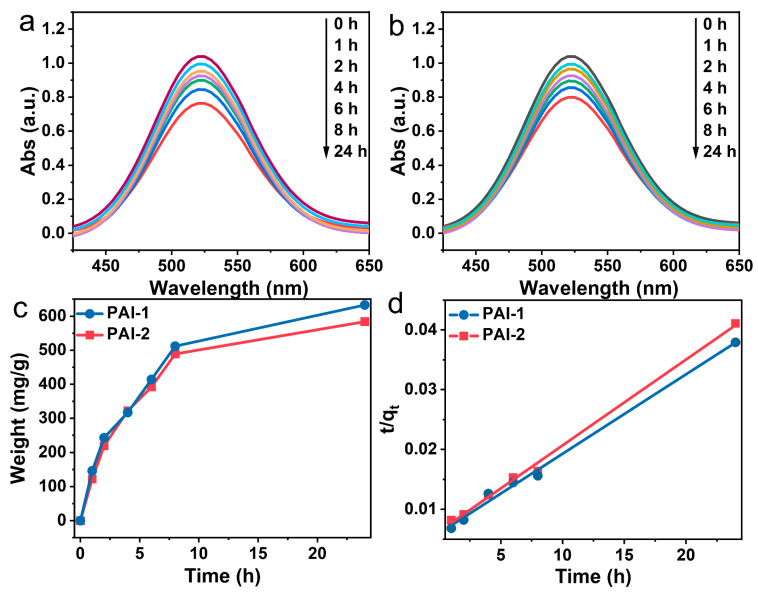
(**a**,**b**) UV-Vis spectra which were recorded during the adsorption experiments of (**a**) PAI-1 and (**b**) PAI-2 in an iodine–cyclohexane solution at various time intervals; (**c**) the adsorption profiles of PAI-1 and PAI-2 in iodine–cyclohexane (0.01 M) solution; (**d**) pseudo-second-order kinetic modeling of the adsorption of iodine–cyclohexane solutions (0.01 mol L^−1^) by PAI-1 and PAI-2.

Considering the presence of numerous electron-withdrawing structural units in the hyperbranched PAIs synthesized in this study, such as trifluoromethyl and acylimine rings, we conducted an adsorption property test on the PAIs. Iodine-129 is a radioactive element that poses risks to the environment and human health, which has garnered strong interest from researchers [46]. Therefore, we evaluated the iodine adsorption performance of the prepared hyperbranched PAIs as follows: 5 mg of each of the two PAIs were placed in an iodine–cyclohexane solution containing 5 mL of iodine with a concentration of 0.01 mL l L^−1^, and the absorbance change of the iodine solution was measured using UV-Vis spectroscopy. Figure 13a,b depict the decrease in absorbance of the iodine solution over time, reaching its minimum value after 24 h. Subsequently, we calculated the adsorption amount using Equation (3):

(3) $$W=[\frac{I_{0}-I_{t}}{I_{0}}\times C\times V]÷m$$

where *I_0_* represents the initial absorbance, *I_t_* represents the absorbance at time *t*, *C* represents the concentration of the iodine solution, *V* represents the volume of the iodine solution, and *m* represents the weight of the sample, resulting in adsorption amounts of 633 mg and 587 mg for hyperbranched PAI 1-2, respectively, over 24 h (Figure 13c).

This result can be attributed to two main factors. First, the lone pair of electrons and unsaturated bonds in the trifluoromethyl and imide rings endow them with electron-absorbing abilities, while iodine typically exists in an ionic form in cyclohexane solution, thus leading to stronger interactions between the polymer and iodine [47,48,49,50]. Second, the high percentage of trifluoromethyl and imide ring units in the polymer structure results in a certain adsorption capacity for iodide ions. This represents the reason for the higher iodine adsorption of PAI-1 compared to PAI-2, as the trifluoromethyl group is also incorporated into its pendant unit group (see in Supplementary Material Figure S14). Additionally, the adsorption process follows pseudo-second-order kinetics, as determined using Equation (4) (Figure 13d):

(4) $$\frac{t}{q_{t}}=\frac{1}{k_{2}q_{e}^{2}}+\frac{t}{q_{e}}$$

where *q_e_* represents the equilibrium adsorption amount, *q_t_* represents adsorption amount of the adsorbent at time *t*, and *k*_2_ represents the pseudo-second-order rate constant.

**Figure 14:**
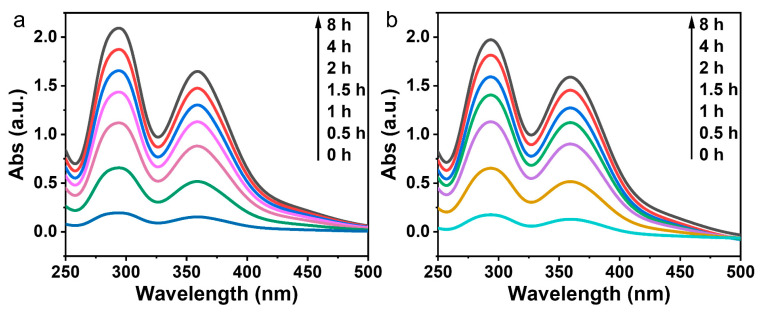
(**a**) UV-Vis spectra which were recorded during the desorption experiments of PAI-1 in an iodine-ethanol solution at various time intervals; (**b**) UV-Vis spectra which were recorded during the desorption experiments of PAI-2 in an iodine-ethanol solution at various time intervals.

Following the adsorption test, we conducted desorption experiments by placing the tested polymers in anhydrous ethanol and measuring the absorbance of the solution via UV-visible spectroscopy. It was observed that the absorbance of the iodine–ethanol solution increased over time, indicating that iodine was released from the polymers (Figure 14).

## 3. Conclusions

In summary, this paper presents a novel triacid monomer that was synthesized by reacting the triamino monomer 1,3,5-tris(4-amino-2-trifluoromethylphenoxy)benzene (TFAPOB) with trimellitic anhydride through a thermally cyclization reaction. The resulting monomers were used to prepare a series of hyperbranched poly(amide-imide)s (PAI-1 and PAI-2) with pendant units and electrochromic properties. These hyperbranched PAIs exhibit excellent solubility and thermal stability. Cyclic voltammetry (CV) analysis confirms the PAI films’ favorable redox properties. Under applied voltage, the color of the PAI films changes from light yellow in the initial state to green and eventually to blue-black. Notably, the coloring efficiency (CE) and transmittance variation (∆T, 852 nm) of PAI-1 were 257 cm^2^/C and 62%, respectively, and long-lasting redox properties were observed. In addition, the verification of the presence of the radical cation N^+^ in polymers after electrochromic behavior was performed via XPS. These results highlight the potential of the synthesized PAIs with electrochromic properties for aerospace applications. Finally, by leveraging the structural features of incorporating electron-absorbing units within the polymers, we conducted iodine adsorption tests. The results demonstrated that the polymers possess a measurable iodine adsorption capacity (633 mg/g), thereby contributing to the advancement of adsorbent technology in the future.

## Figures and Tables

**Table 1:** Thermal properties of PAI-1 and PAI-2.

Polymers	DSC	TGA		
	T ^a^ (°C)	T_5%_ ^b^ (°C)	T_10%_ ^c^ (°C)	Char Yied ^d^ (%)
PAI-1	256	574	608	56.2
PAI-2	261	514	564	60.7

^a^ Glass transition temperatures recorded at a heating rate of 20 °C/min under a nitrogen atmosphere. ^b^ The polymers were heated at a rate of 10 °C/min until a weight loss of 5% was produced, and the temperature was recorded via TGA under a nitrogen atmosphere. ^c^ The polymers were heated at a rate of 10 °C/min until a weight loss of 5% was produced, and the temperature was recorded via TGA under a nitrogen atmosphere. ^d^ Residual weight (%) when heated to 800 °C.

**Table 2:** Inherent viscosity, GPC values, and solubility of the PAIs.

Polymers	Inherent Viscosity	GPC Data		Solvents							
	n_inh_ ^a^ (dL/g)	M_w_ ^b^ (g/mol)	M_w_/M_n_ ^b^	DMSO	NMP	DMAc	DMF	THF	CHCl_3_	EtOAc	EtOH
PAI-1	0.41	6.2 × 10^4^	2.3	++	++	++	++	+	-	-	-
PAI-2	0.43	6.5 × 10^4^	2.1	++	++	++	++	+	-	-	-

^a^ Determined with 0.5% solutions in a solvent (DMAc) at 25 °C. ^b^ Relative to a polystyrene standard, using DMF as the eluent. Qualitative solubility test using 10 mg/mL as the solubility benchmark. ++: Fully dissolved at room temperature. +: Partially soluble at room temperature. -: Not soluble even when heated.

**Table 3:** Electrochemical properties of the PAIs.

Index	Thin Film (λ/nm)		Oxidation Potential ^a^ (V)			E_g_^b^ (eV)	HOMO ^c^ (eV)	LUMO ^d^ (eV)
	Abs.max	Abs.onset	Eonset	E_OX1_1/2	E_OX2_1/2			
PAI-1	337	406	0.61	1.16	1.71	3.05	4.83	1.78
PAI-2	337	391	0.63	1.07	1.78	3.17	4.85	1.68

**^a^** Oxidation half-wave potentials from cyclic voltammograms. **^b^** The data were calculated by the equation: *E*_g_ = 1240/λ_onset_ of polymer film. **^c^** The HOMO energy levels were calculated from cyclic voltammetry and were referenced to ferrocene (4.8 eV). **^d^** LUMO = HOMO − *E*_g_.

## Data Availability

Data are contained within the article.

## Supplementary Materials

The following supporting information can be downloaded at: https://www.mdpi.com/article/10.3390/polym15234537/s1.
