# Supplementary material for: Preparation of Novel Nitrogen-Rich Fluorinated Hyperbranched Poly(amide-imide) and Evaluation of Its Electrochromic Properties and Iodine Adsorption Behavior
Source: Polymers (Basel). 2023 Nov 25;15(23):4537. doi: 10.3390/polym15234537 (PMC10707875; doi:10.3390/polym15234537)
Supplement: Supplementary file 1 [file polymers-15-04537-s001.zip › polymers-2700484-supplementary.pdf]

Supplementary Materials

Preparation of Novel Nitrogen-Rich Fluorinated Hyperbranched Poly(amide-imide) and Evaluation of Its Electrochromic Properties and Iodine Adsorption Behavior

Zebang Sun [1], Wen Yang [1], Xiaosa Zhang [1], Xiaoyu Zhu [1], Jian Luan [2], Wenze Li [1,*] and Yu Liu [1,*]

[1] College of Science, Shenyang University of Chemical Technology, Shenyang 110142, China; sunzebang0826@163.com (Z.S.); wenyang120105@163.com (W.Y.); xszhang@syuct.edu.cn (X.Z.); zhuxiaoyu@syuct.edu.cn (X.Z.)

[2] College of Sciences, Northeastern University, Shenyang 110819, China; 2010044@stu.neu.edu.cn

* Correspondence: liwenze@syuct.edu.cn (W.L.); liuyu@syuct.edu.cn (Y.L.)

Contents

1. **Experiment section** (P 2)
2. **Figure S1.** ^{1}H NMR spectrum of TFAPOB in DMSO-d_6. (P 7)
3. **Figure S2.** FT-IR spectra of the TFNPOB and TFAPOB. (P 8)
4. **Figure S3.** ^{1}H NMR spectrum of TCA-F in DMSO-d_6. (P 9)
5. **Figure S4.** FT-IR spectra of the TCN-F and TCA-F. (P 10)
6. **Figure S5.** ^{1}H NMR spectrum of TCIA in DMSO-d_6. (P 11)
7. **Figure S6.** FT-IR spectra of the TCIN and TCIA. (P 12)
8. **Figure S7.** FT-IR spectra of the TFAPOB and BTTFTDCA. (P 13)
9. **Figure S8.** UV–Vis spectra of the polymers of PAI-1 and PAI-2 in NMP solution. (P 14)
10. **Figure S9.** The XRD spectrogram of PAI-1 and PAI-2. (P 15)
11. **Figure S10.** Tapping-mode AFM topography image of the PAI-1 film (4 μm × 4 μm). (P 15)
12. **Figure S11.** Tapping-mode AFM topography image of the PAI-2 film (4 μm × 4 μm). (P 16)
13. **Figure S12.** Plot of current versus time of PAI-1 and PAI-2 during electrochromic behavior in 4s. (P 16)
14. **Figure S13.** PAI-1 and PAI-2 reversible electrochromic optical images. (P 17)
15. **Figure S14.** Diagram of the adsorption mechanism of iodine by PAIs. (P 18)
16. **Reference** (P 18)

1. Experiment section

1.1. Materials

The materials employed in this investigation were as follows: anhydrous phloroglucinol (99%, Macklin), 2-chloro-5-nitrobenzotrifluoride (98%, J&K Chemicals), anhydrous potassium carbonate (99%, Sinopharm), toluene (99%, Sinopharm), *N*,*N*-dimethylformamide (99%, Sinopharm, DMF), methanol (99%, Sinopharm), palladium on carbon hydrogenation 10% catalyst (10%, J&K Chemicals), hydrazine hydrate (85%, Sinopharm), ethanol (99%, Sinopharm, EtOH), acetic acid (99%, Sinopharm), trimellitic anhydride (97%, Macklin), carbazole (98%, Macklin), p-fluoronitrobenzene (99%, Macklin), *N*-methylpyrrolidone (99%, Sinopharm, NMP), anhydrous calcium chloride (98%, Macklin), Triphenyl phosphite (98%, Macklin, TPP), pyridine (99%, Sinopharm, Py), *N*,*N*-dimethylacetamide (99%, Sinopharm, DMAc), chloroform (99%, Sinopharm), tetrahydrofuran (99%, Sinopharm, THF), dimethyl sulfoxide (99%, Sinopharm, DMSO), dichloromethane (99%, Sinopharm, DCM), ethyl acetate (99%, Sinopharm, EtOAc), anhydrous acetonitrile (99%, Sinopharm) tetrabutylammonium perchlorate (98%, Macklin), and potassium chloride (99%, Sinopharm).

1.2. Monomer synthesis

1.2.1. Synthesis of 1,3,5-tris(4-nitro-2-(trifluoromethyl)-phenoxy)-benzene (TFNPOB, monomer 1)

As shown in **Scheme S1**, the synthesis of TFNPOB was performed in reference to previously reported methods [37]. In a 500 mL three-necked round-bottomed flask, 32.0 g (232 mmol) of anhydrous potassium carbonate, 6.3 g (50 mmol) of anhydrous phloroglucinol, and 35.5 g (158 mmol) of 2-chloro-5-nitrobenzotrifluoride were added into a solution which consisted of DMF (150 mL) and toluene (80 mL) under a nitrogen atmosphere. The flask was connected a Dean–Stark trap, and we added another portion of toluene to the trap branch pipe, after which we heated the mixture to 135 ℃ and refluxed for 4 hours, and then evaporated the toluene from the system. Then, we raised the temperature to 150 ℃, and continued the reaction for 8 hours. After 8 hours, we poured the mixture into the ice–water mixture, filtered and collected the solid product, washed it with distilled water 8 times and methanol 4 times, and finally dried it in a 60 ℃ vacuum drying oven to obtain a yellow solid powder (TFNPOB). T_m: 160 – 163 ℃. FTIR (KBr): 1520 and 1340 cm^{-1} ($-NO_2$ stretching), 1127 cm^{-1} ($-CF_3$ stretching). ^1H NMR (500 MHz, DMSO-d_6) δ = 7.43 (t, 3H), 7.87 (t, 3H), 7.97 (d, 3H), 8.39 (d, 3H). Elem. Anal. Calcd of $C_{27}H_{12}F_9N_3O_9$ (693.4): C, 46.77; H, 1.74; N, 6.06. Found: C, 46.97; H, 1.58; N, 6.02.

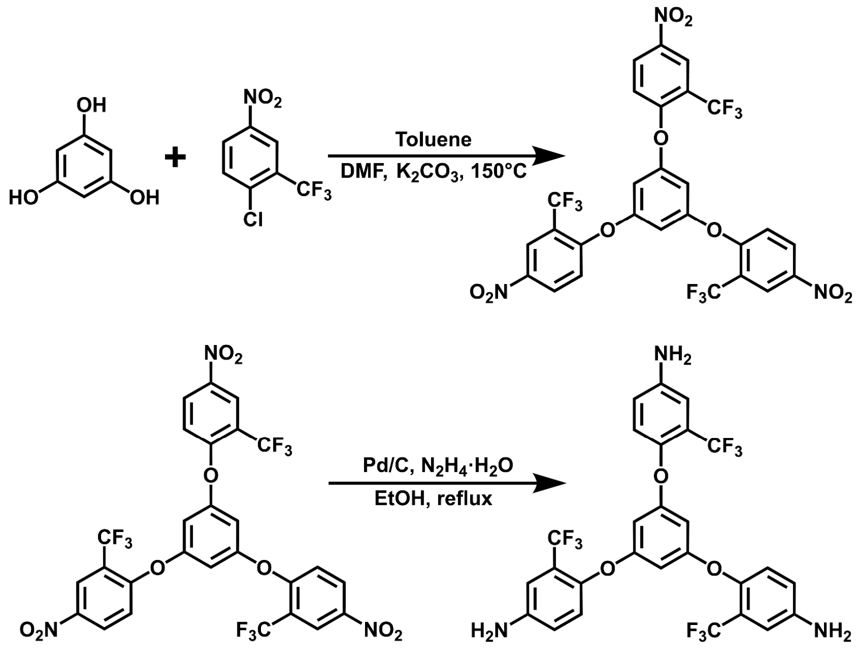

Scheme S1. Synthetic route towards TFAPOB.

1.2.2. Synthesis of 1,3,5-tris(4-amino-2-(trifluoromethyl)phenoxy)-benzene (TFAPOB, monomer 2)

In a 250mL three-necked round-bottomed flask, TFNPOB (6.94g, 1mmol) and Pd/C (10%, 0.20g) were dispersed in ethanol (100mL), and then hydrazine hydrate (85%) was slowly dropped under a nitrogen atmosphere. Afterwards, we refluxed the mixture for 3 days. We removed Pd/C through thermal filtration through a sand core funnel, and concentrated the filtrate through rotary evaporation to remove excess hydrazine hydrate. Under stirring, the mixture was placed in cooled distilled water to obtain a light-yellow precipitate. We filtered and collected the formed product, thoroughly washed it with distilled water five times, vacuum-dried it, and purified it by means of dichloromethane (DCM) column chromatography elution to produce a white solid powder (**Scheme S1**). T_m: 179 – 181 ℃. FTIR (KBr): 3430 and 3160 cm^{-1} ($-NH_2$ stretching), 1127 cm^{-1} ($-CF_3$ stretching). ^1H NMR (500 MHz, DMSO-d_6) δ = 6.90 (d, *J* = 8.8 Hz, 1H), 6.86 (d, *J* = 2.2 Hz, 1H), 6.83 – 6.70 (m, 1H), 5.97 (s, 1H), 5.48 (s, 2H). Elem. Anal. Calcd of $C_{27}H_{18}F_9N_3O_3$ (603.4): C, 53.74; H, 3.01; N, 6.96. Found: C, 53.92; H, 3.11; N, 6.68.

1.2.3. Synthesis of 2,2',2''-((benzene-1,3,5-triyltris(oxy))tris(3-(trifluoromethyl)benzene-4,1-diyl))tris(1,3-dioxoisoindoline-5-carboxylic acid) (BTTFTDCA, monomer 3)

In a 50 mL three-necked round-bottomed flask, TFAPOB 3.2 g (5 mmol) and trimellitic anhydride 2.9 g (15 mmol) were dispersed in acetic acid (18 mL) under a nitrogen atmosphere. Afterwards, we refluxed the mixture for 12 hours. After 12 hours, we turned off the heating and stirring devices, let the product stand still, and filtered it the next day to obtain the triacid monomer. T_m: 269 – 271 ℃. FTIR (KBr): 3434.16 cm^{-1} ~ 2538.87 cm^{-1} (-OH stretching), 1697.14cm^{-1} (-C=O stretching), 1293.30cm^{-1} (C-N stretching), 1127 cm^{-1} ($-CF_3$ stretching). ^1H NMR (500 MHz, DMSO-d_6) δ = 8.45 – 8.37 (m, 1H), 8.26 (s, 1H), 8.06 (t, *J* = 7.6 Hz, 1H), 7.94 (d, *J* = 2.0 Hz, 1H), 7.80 (dd, *J* = 8.8, 1.9 Hz, 1H), 7.47 (d, *J* = 8.9 Hz, 1H), 6.81 (s, 1H). Elem. Anal. Calcd of $C_{54}H_{24}F_9N_3O_{15}$ (1125.8): C, 57.61; H, 2.15; N, 3.73. Found: C, 57.91; H, 2.04; N, 3.54.

1.2.4. Synthesis of **3**′′-trifluoromethyl-4,4′-diamino-4′′-N-carbazolyl triphenylamine (TCA-F, monomer 4)

TCA-F was synthesized following a previously reported procedure (**Scheme S2**) [32]. Carbazole, 4-fluoronitrobenzene, and 2-chloro-5-nitro-trifluorotoluene were employed as

reactive monomers in a multistep C-N coupling reaction and a subsequent hydrazine hydrate reduction reaction to obtain the diamine monomer TCA-F. The characterization data of the TCA-F diamine monomer are presented below: T_m: 114–116 ℃. FT-IR (KBr, cm^{-1}): 3511, 3451 (NH_2 stretching). ^1H NMR (500 MHz, DMSO-d_6, δ, ppm): 8.19 (t, J = 15.4 Hz, 2H), 7.38 (dd, J = 17.5, 15.5 Hz, 2H), 7.23 (t, J = 7.4 Hz, 2H), 7.12 (t, J = 13.4 Hz, 1H), 7.03 (t, J = 12.8 Hz, 4H), 6.98 (d, J = 7.8 Hz, 2H), 6.97 (s, 1H), 6.89 (dd, J = 8.8, 2.6 Hz, 1H), 6.63 (d, J = 8.6 Hz, 4H), 5.17 (s, 4H, –NH_2). 13C NMR (126 MHz, DMSO-*d*6, δ, ppm): 151.05, 147.45, 143.89, 134.93, 133.02, 128.74, 126.47, 124.76, 122.91, 122.57, 120.68, 120.11, 119.25, 115.55, 112.25, 110.43. Elem. Anal. Calcd of $C_{31}H_{23}F_3N_4$ (508.6): C, 73.22; H, 4.56; N, 11.02. Found: C, 72.89; H, 4.64; N, 11.27.

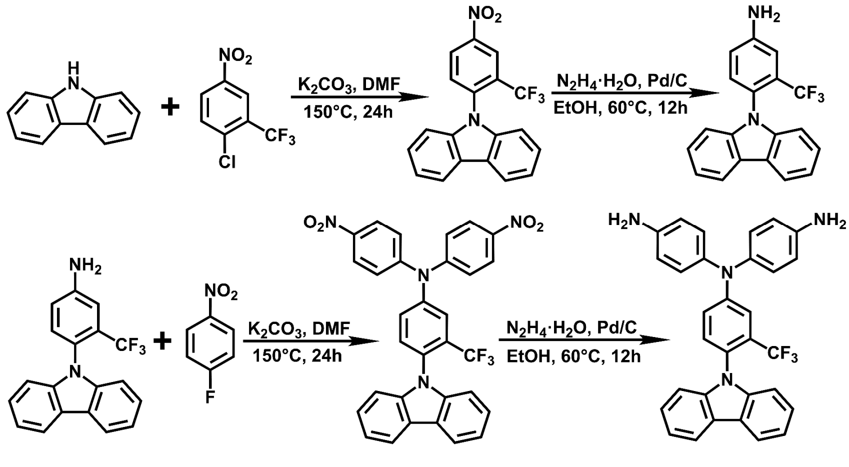

Scheme S2. Synthetic route towards TCA-F.

1.2.5. **Synthesis of** 4,4′-bis[(4-aminophenyl)amino]-4′′-carbazolyltriphenylamine (TCIA, monomer 5)

TCIA was synthesized following a previously reported procedure (**Scheme S3**) [33]. Carbazole and 4-fluoronitrobenzene were employed as reactive monomers in a multistep C-N coupling reaction and subsequent hydrazine hydrate reduction reaction to obtain the diamine monomer TCIA. The characterization data of the TCIA diamine monomer are presented below: T_m: 120–122 ℃, FT-IR (KBr, cm^{-1}): 3439, 3368 (NH_2 stretch). ^1H NMR (DMSO-*d*6, 500 MHz, δ, ppm): 8.19 (2H, d, J = 7.8 Hz),7.49 (2H, s), 7.39 (2H, t, J = 7.6 Hz), 7.27 (6H, m), 7.04 (2H, d, J = 8.6 Hz), 6.84 (12H, m), 6.55 (4H, d, J = 8.4 Hz), 4.74 (4H, s). ^{13}C NMR (DMSO-d_6, 126 MHz, δ, ppm): 149.17, 144.49, 144.26, 141.18, 137.04, 132.30, 128.05, 127.80, 127.17, 136.37, 122.95, 122.83, 120.82, 119.95, 117.90, 115.33, 110.03. Elem. Anal. Calcd of $C_{42}H_{34}N_6$ (622.8): C, 81.00; H, 5.50; N, 13.49. Found: C, 80.66; H, 5.54; N, 13.79.

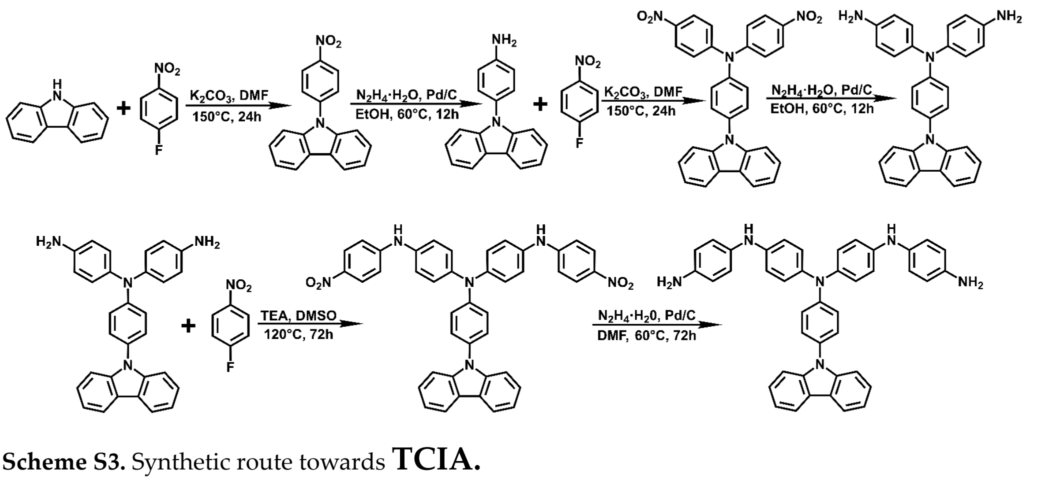

Scheme S3. Synthetic route towards **TCIA.**

1.3. Polymer synthesis

1.3.1. Synthesis of PAI-1

As shown in **Scheme S4**, BTTFTDCA (monomer 3, 0.1126 g, 0.1 mmol) and TCA-F (monomer 4, 0.0509 g, 0.1 mmol) were dissolved in 1 mL of NMP, respectively, and then were added into a three-necked flask. After that, 1.0 mL of triphenyl phosphite (TPP), 0.2 g of anhydrous calcium chloride, and 0.5 mL of pyridine were added into the flask. The mixture was heated at 120 ℃ for 4 hours. After 4 hours, the mixture was poured into 300 mL of ethanol with stirring, we filtered and collected the products, and washed them with ethanol three times. A Soxhlet extractor was used to extract the resulting polymer with ethanol for 24 h. After drying in a vacuum, PAI-1 (0.1359 g, yield: 85 %) was obtained. FTIR (KBr): 1727.64 cm^{-1} (C=O stretching), 1237.57 cm^{-1} (C-N stretching), 1135.54 cm^{-1} (-CF_3 stretching). ^1H NMR (500 MHz, DMSO-*d*$_6$) δ = 10.85 – 10.68 (m, 0H), 8.52 (s, 0H), 8.46 (s, 1H), 8.19 (s, 1H), 8.09 (s, 1H), 7.94 (q, *J* = 10.8, 10.3 Hz, 2H), 7.80 (d, *J* = 14.4 Hz, 1H), 7.48 (t, *J* = 9.7 Hz, 1H), 7.39 (d, *J* = 8.2 Hz, 1H), 7.35 (s, 2H), 7.26 (s, 1H), 7.25 (s, 2H), 7.25 – 7.11 (m, 1H), 7.10 – 7.01 (m, 1H), 6.83 (d, *J* = 13.7 Hz, 1H). ^{13}C NMR (DMSO-*d*$_6$, 126 MHz, δ, ppm): 166.74, 163.79, 158.63, 157.74, 153.61, 150.29, 149.10, 142.98, 134.89, 133.85, 132.27, 130.39, 129.73, 128.18, 125.81, 124.49, 124.07, 123.02, 122.71, 122.22, 120.89, 120.11, 119.32, 118.39, 115.57, 110.48, 107.27. Elem. Anal. Calcd: C, 64.99; H, 3.16; N, 6.03. Found: C, 65.31; H, 2.99; N, 5.88.

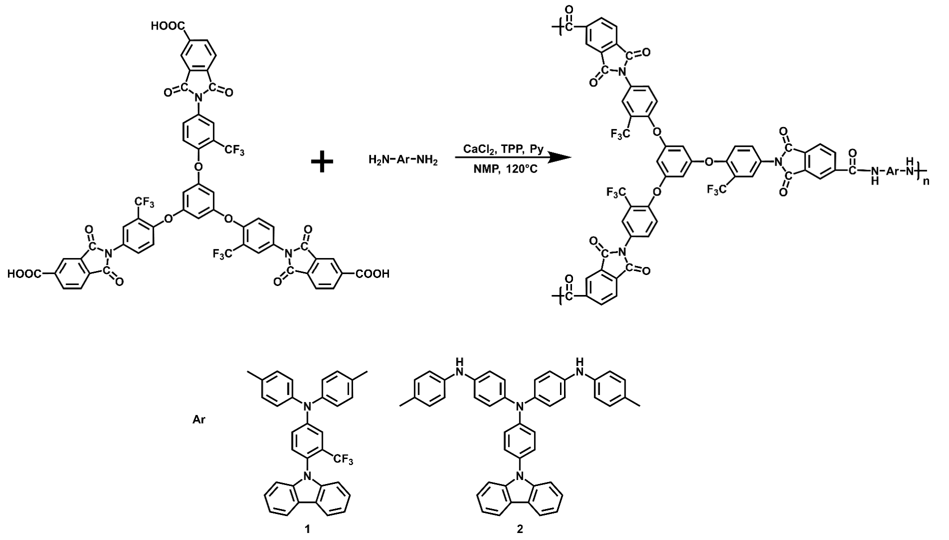

Scheme S4. Synthetic route towards the polymers PAI-1 and PAI-2.

1.3.2. Synthesis of PAI-2

The BTTFTDCA (monomer 3, 0.1126 g, 0.1 mmol) and TCIA (monomer 5, 0.0623 g, 0.1 mmol) were dissolved in 1 mL of NMP, respectively, and then were added into a three-necked flask. After that, 1.0 mL of triphenyl phosphite (TPP), 0.2 g of anhydrous calcium chloride, and 0.5 mL of pyridine were added into the flask. The mixture was heated at 120 ℃ for 4 hours. After 4 hours, the mixture was poured into 300 mL of ethanol with stirring, we filtered and collected the products, and washed them with ethanol three times. A Soxhlet extractor was used to extract the resulting polymer with ethanol for 24 h. After drying in a vacuum, PAI-2 (0.1422 g, yield: 83 %) was obtained (**Scheme S4**). FTIR (KBr): 1727.57 cm^{-1} (C=O stretching), 1238.42 cm^{-1} (C-N stretching). ^1H NMR (500 MHz, DMSO-*d*$_6$) δ = 8.49 (d, *J* = 19.4 Hz), 8.17 (d, *J* = 29.7 Hz), 7.96 (d, *J* = 13.2 Hz), 7.80 (d, *J* = 8.5 Hz), 7.71, 7.46 (dd, *J* = 11.6, 5.4 Hz), 7.42 – 7.31 (m), 7.27 (t, *J* = 6.3 Hz), 7.21 – 7.01 (m), 6.89 – 6.68 (m). ^{13}C NMR (DMSO-*d*$_6$, 126 MHz, δ, ppm): 166.90, 157.95, 153.39, 150.27, 140.95, 136.68, 133.82, 132.27, 130.88, 130.56, 129.81, 128.19, 125.72, 124.16, 122.81, 120.72, 119.08, 115.73, 110.23, 107.05. Elem. Anal. Calcd: C, 68.31; H, 3.59; N, 7.24. Found: C, 68.54; H, 3.42; N, 7.18.

1.4. Characterization

Solution ^{1}H NMR spectroscopy was performed with a Bruker Ascend 500 MHz spectrometer. Solution ^{13}C NMR spectroscopy was performed with a Bruker Ascend 126 MHz spectrometer. Cyclic voltammetry measurements were performed using a three-electrode cell. All cell potentials were recorded using ITO (with a polymer film area of~0.8 cm × 1.5 cm) as the working electrode, a platinum wire as the auxiliary electrode, and Ag/AgCl (with a saturated KCl solution) as a homemade reference electrode. The voltammograms were presented with the potential arranged to the left and with increasing anodic currents directed downward. The spectroelectrochemical cell consisted of a 1 cm cuvette, ITO as a working electrode, a platinum wire as an auxiliary electrode and Ag/AgCl as the reference electrode. Absorption spectra were recorded using a Shimadzu UV 3101-PC spectrophotometer. Fourier transform infrared spectroscopy (FT-IR) spectra were recorded using a Nicolet Impact 410 Fourier-transform infrared spectrometer. Elemental analysis was performed using an Elemental Analyses MOD-1106 system. The inherent viscosity was measured with an Ubbelohde viscometer that was fixed in a thermostatic container at 25 °C, and the concentration of the polymer solution in dimethylacetamide (DMAc) was 0.5 dL g^{-1}. Digital security control (DSC) measurements were conducted using a Mettler Toledo DSC 821e instrument at a heating rate of 20 °C min^{-1} in a nitrogen atmosphere. Thermogravimetric analyzer (TGA) measurements were performed in a nitrogen atmosphere at a heating rate of 10 °C min^{-1} and the PAIs were loaded in open aluminum pans equipped with a PerkinElmer TGA-7 calorimeter. Tensile tests were performed using a Shimadzu AG-I system at a constant crosshead speed of 10 mm min^{-1}. The chromaticity of each film was measured using a PR-655 spectrophotometer (American company). Wide-angle X-ray diffraction (WAXD) measurements were performed at room temperature using a Rigaku/max-rA diffractometer that was equipped with a Cu Kα radiation source. Gel permeation chromatograms (GPC) were obtained on a Waters 410 instrument with N, N-dimethylformamide (DMF) as an eluent at a flow rate of 1 mL min^{-1} using polystyrene as a standard. Photoluminescence spectra were measured with a Jasco FP-6300 spectrofluorometer. X-ray photoelectron spectroscopy (XPS) was performed using an Escalab 250 instrument with Al Kα radiation.

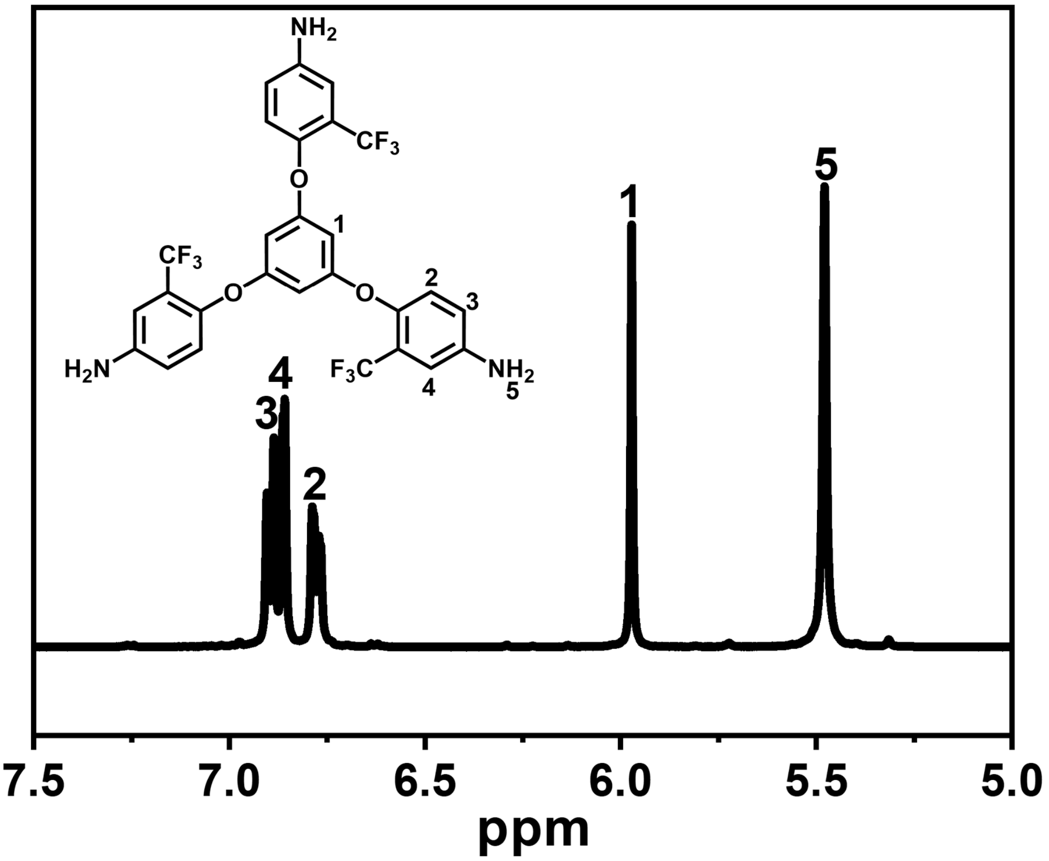

Figure S1. ^{1}H NMR spectrum of TFAPOB in DMSO-d_6.

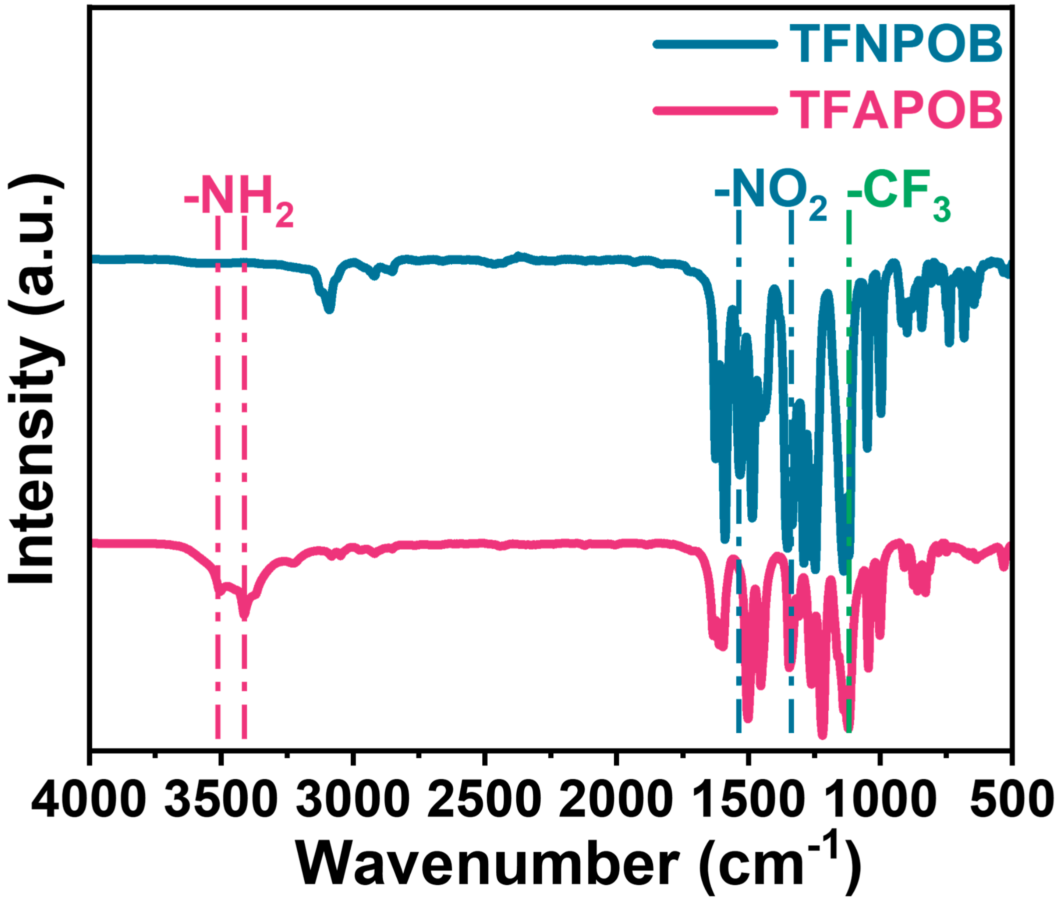

Figure S2. FT-IR spectra of the TFNPOB and TFAPOB.

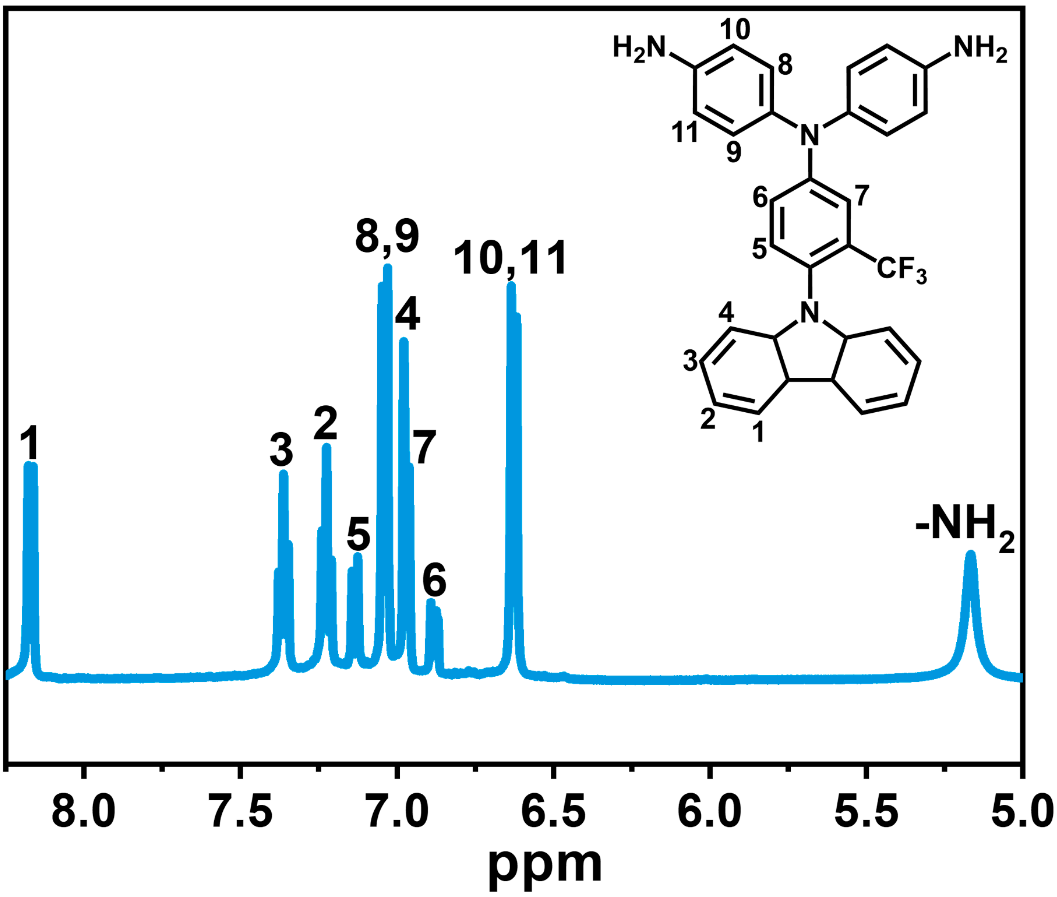

Figure S3. ¹H NMR spectrum of TCA-F in DMSO-*d*₆.

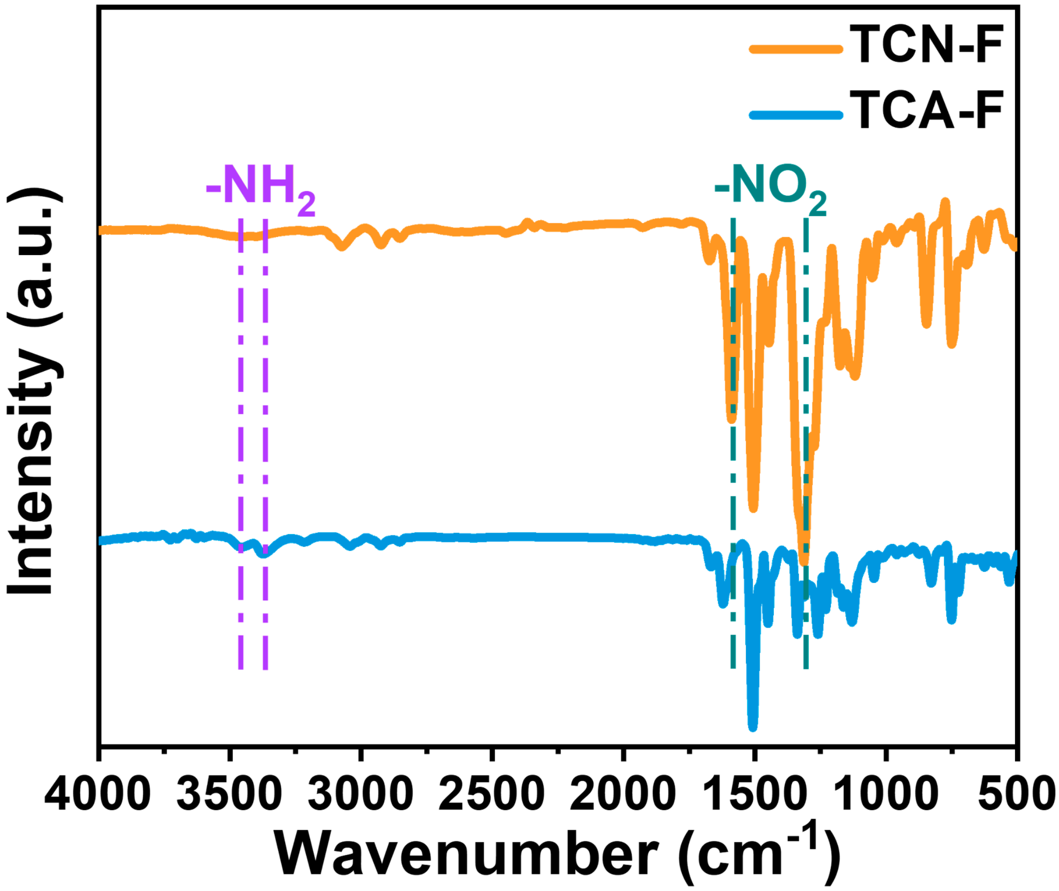

Figure S4. FT-IR spectra of the TCN-F and TCA-F.

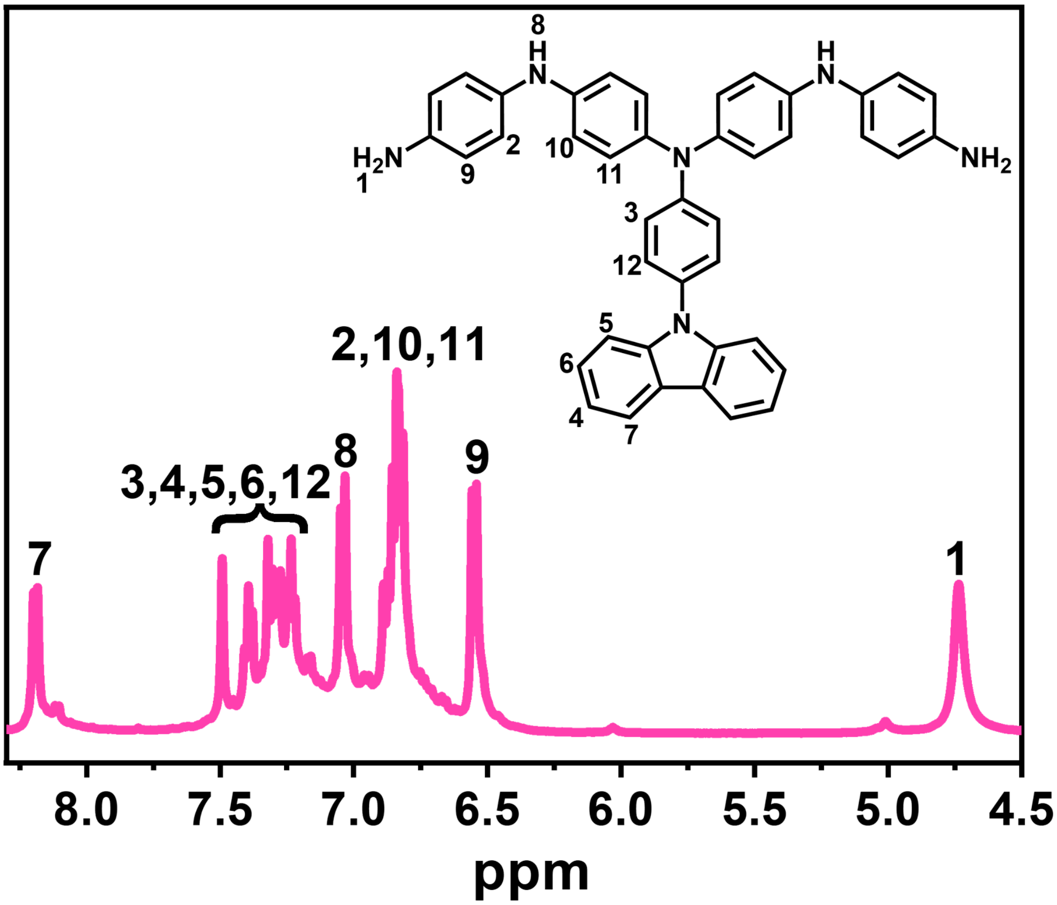

Figure S5. ^{1}H NMR spectrum of TCIA in DMSO-d_6.

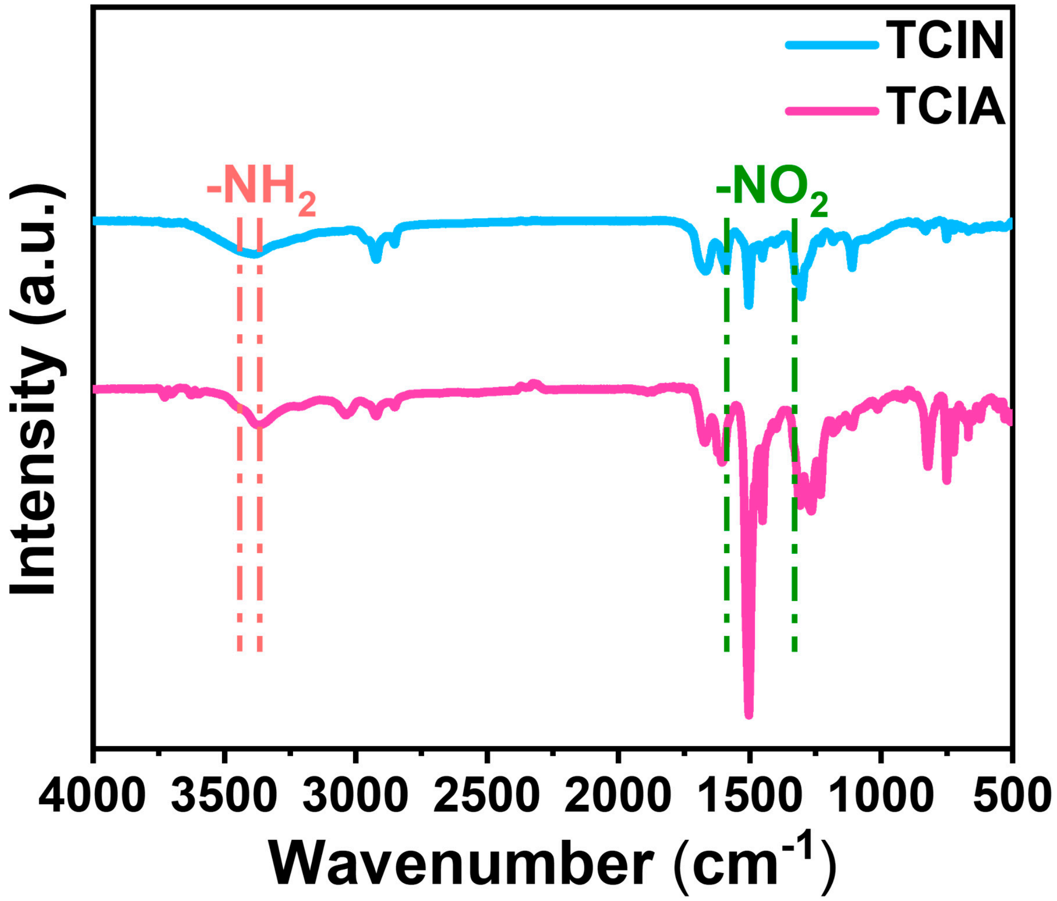

Figure S6. FT-IR spectra of the TCIN and TCIA.

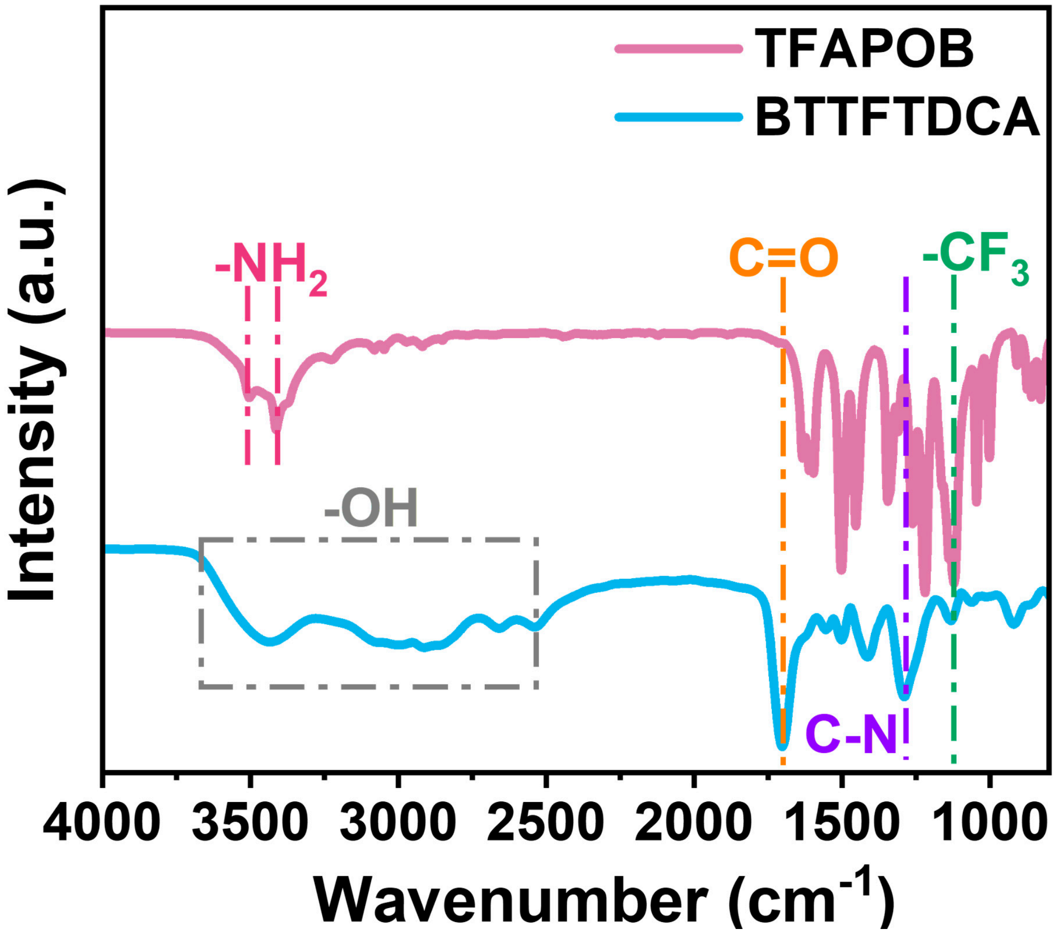

Figure S7. FT-IR spectra of the TFAPOB and BTTFTDCA.

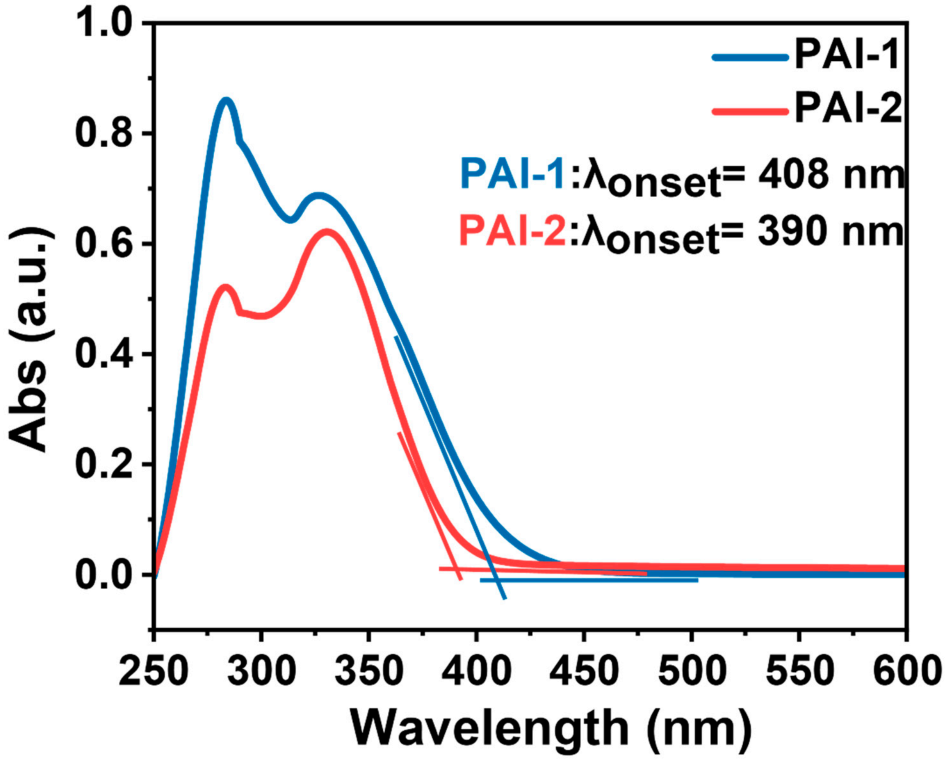

Figure S8. UV–Vis spectra of the polymers of PAI-1 and PAI-2 in NMP solution.

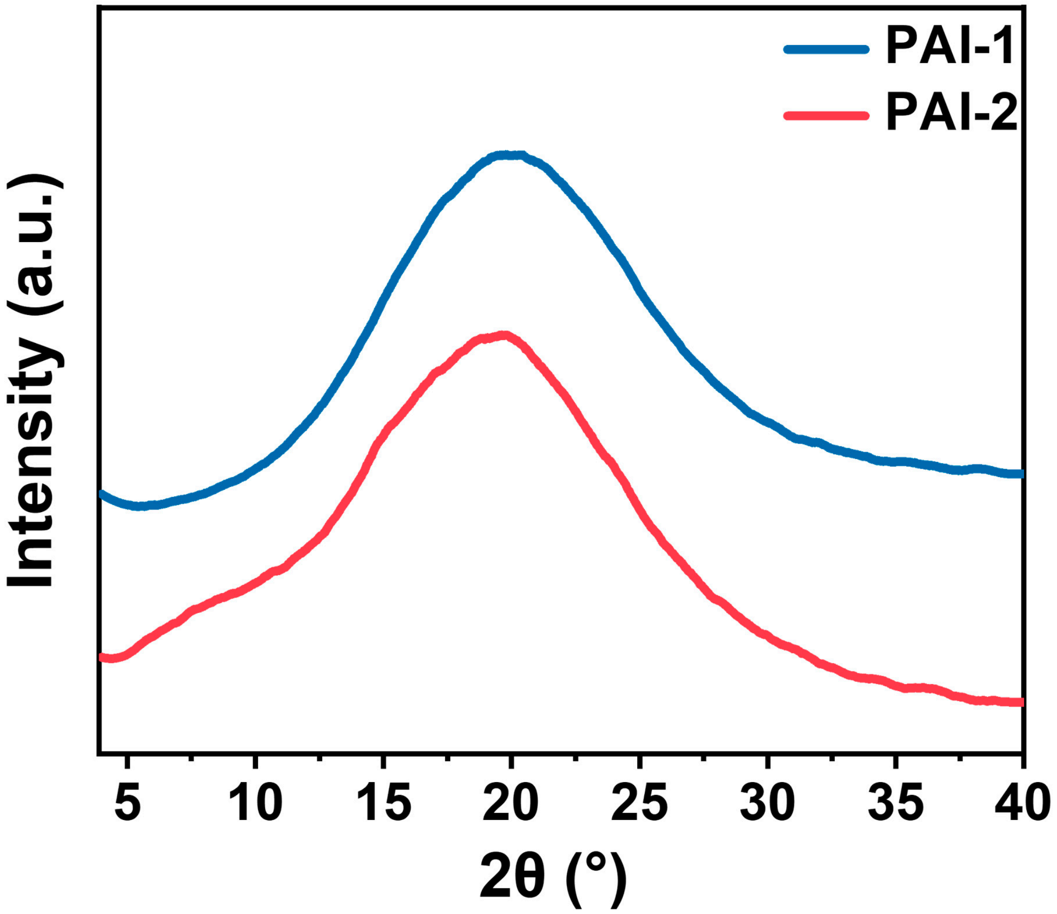

Figure S9. The XRD spectrogram of PAI-1 and PAI-2.

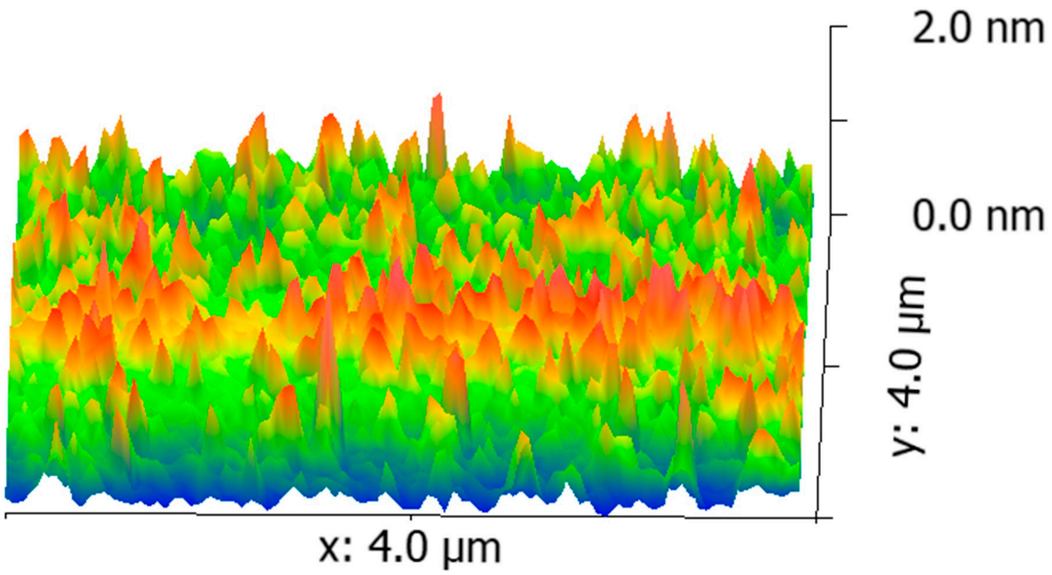

Figure S10. Tapping-mode AFM topography image of the PAI-1 film (4 µm × 4 µm).

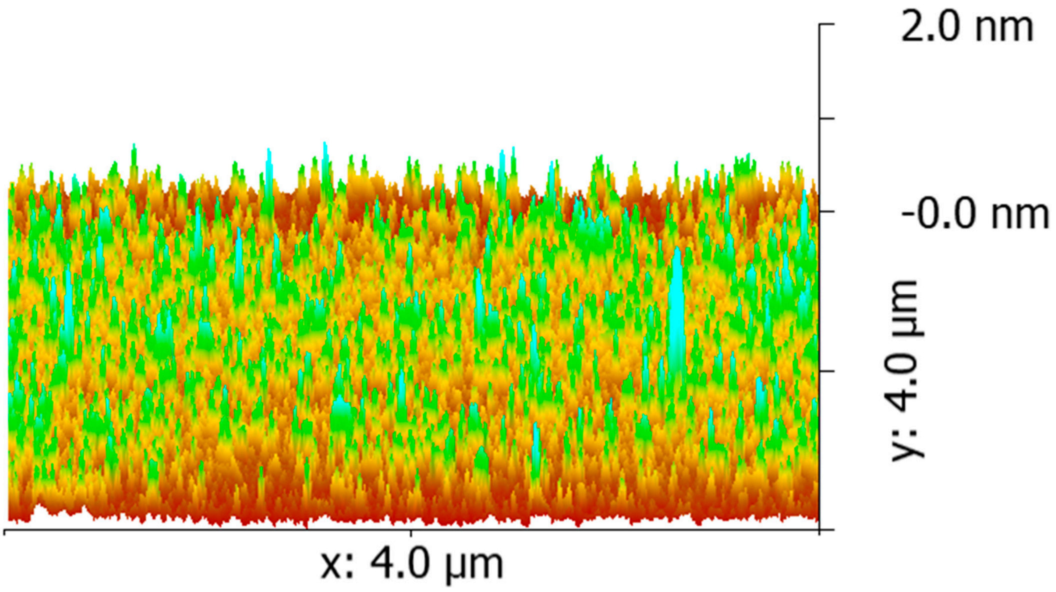

Figure S11. Tapping-mode AFM topography image of the PAI-2 film (4 µm × 4 µm).

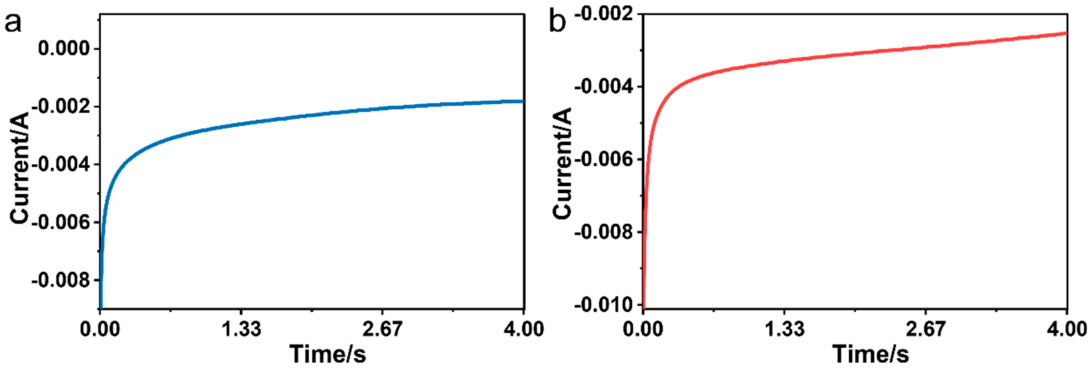

Figure S12. Plot of current versus time of PAI-1 and PAI-2 during electrochromic behavior in 4s.

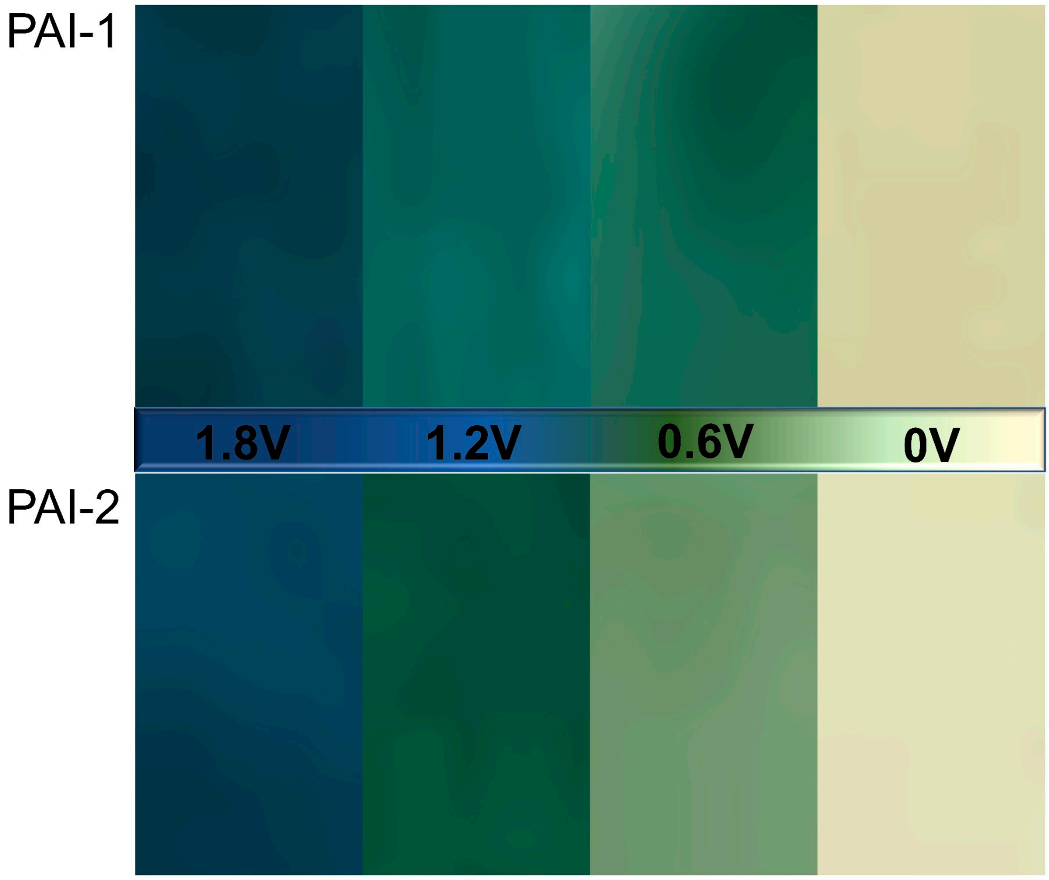

Figure S13. PAI-1 and PAI-2 reversible electrochromic optical images.

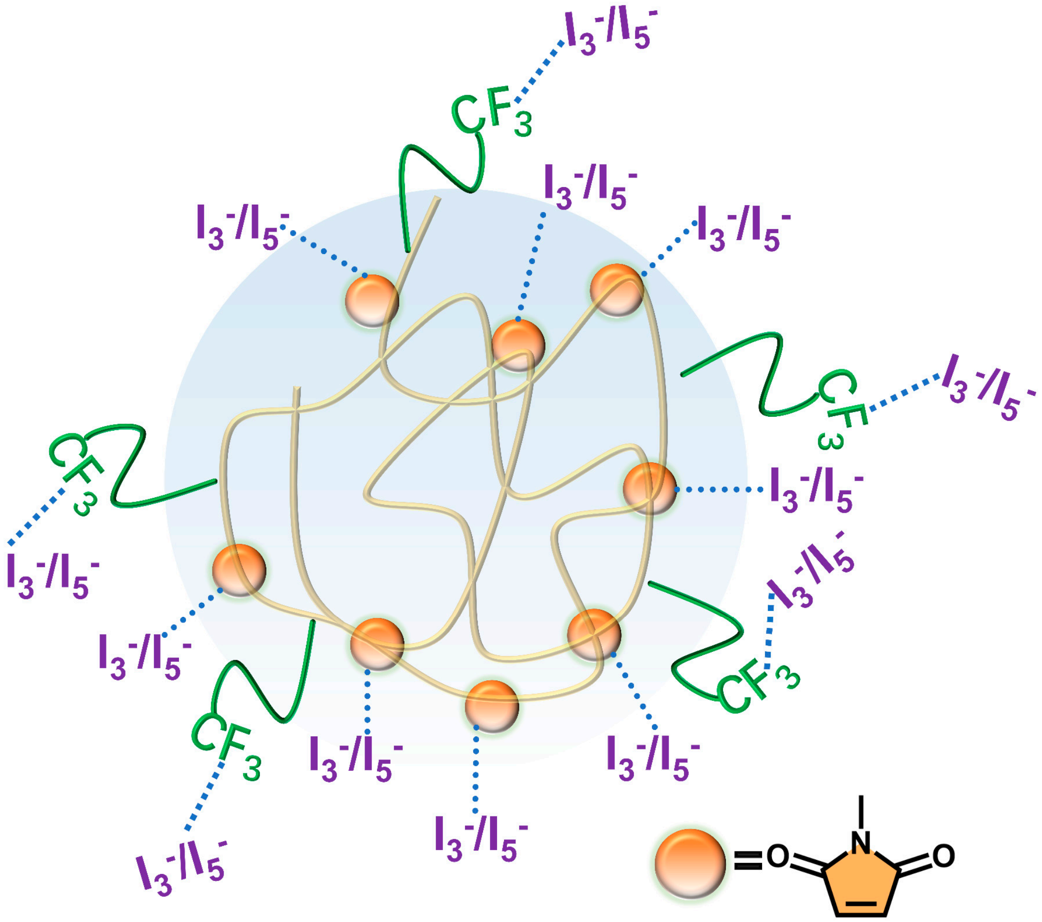

Figure S14. Diagram of the adsorption mechanism of iodine by PAIs.
